# *Bacillus subtilis* PcrA Helicase Removes Trafficking Barriers

**DOI:** 10.3390/cells10040935

**Published:** 2021-04-17

**Authors:** María Moreno-del Álamo, Begoña Carrasco, Rubén Torres, Juan Carlos Alonso

**Affiliations:** Department of Microbial Biotechnology, Centro Nacional de Biotecnología, CNB-CSIC, 28049 Madrid, Spain; mmoreno@cnb.csic.es (M.M.-d.Á.); bcarrasc@cnb.csic.es (B.C.); rtorres@cnb.csic.es (R.T.)

**Keywords:** replication fork stalling, RNA polymerase backtracking, replication–transcription conflict, R-loops

## Abstract

*Bacillus subtilis* PcrA interacts with the RNA polymerase and might contribute to mitigate replication–transcription conflicts (RTCs). We show that PcrA depletion lethality is partially suppressed by *rnhB* inactivation, but cell viability is significantly reduced by *rnhC* or *dinG* inactivation. Following PcrA depletion, cells lacking RnhC or DinG are extremely sensitive to DNA damage. Chromosome segregation is not further impaired by *rnhB* or *dinG* inactivation but is blocked by *rnhC* or *recA* inactivation upon PcrA depletion. Despite our efforts, we could not construct a Δ*rnhC* Δ*recA* strain. These observations support the idea that PcrA dismantles RTCs. Purified PcrA, which binds single-stranded (ss) DNA over RNA, is a ssDNA-dependent ATPase and preferentially unwinds DNA in a 3′→5′direction. PcrA unwinds a 3′-tailed RNA of an RNA-DNA hybrid significantly faster than that of a DNA substrate. Our results suggest that a replicative stress, caused by mis-incorporated rNMPs, indirectly increases cell viability upon PcrA depletion. We propose that PcrA, in concert with RnhC or DinG, contributes to removing spontaneous or enzyme-driven R-loops, to counteract deleterious trafficking conflicts and preserve to genomic integrity.

## 1. Introduction

RNA polymerase (RNAP) transcription elongation complex is likely to encounter protein roadblocks and DNA lesions much more frequently than the replicative DNA polymerase (DNAP) moving along the same DNA template [1,2,3,4]. These roadblocks cause a transient slowing-down or stalling of the replication forks, leading to a replication stress. By contrast, a direct coupling of transcription and translation, with the ribosomes trailing to the nascent mRNA strand as it emerges from the RNAP complex, gives rise to an extremely stable RNAP elongation complex that hinders the lesion and ensures that the outgoing nascent RNA does not become entangled with the DNA helix [1,2,4,5,6].

When a stalled DNA replication fork clashes with a transcription elongation complex the paused transcription elongation complex has a strong tendency to reverse, situation referred to as RNAP backtracking. Here, the altered topological states of the region may facilitate an inappropriate re-hybridization of the nascent transcript with the open duplex DNA behind the transcription complex, displacing the non-coding single-stranded (ss) DNA and leading to the formation of a displacement RNA loop (R-loop) [2,4,5]. These R-loops act as a replication or RNAP elongation block, and can help to transcription termination or to the initiation of DNA replication [3,4,5,6,7].

During unperturbed bacterial growth, an encounter of the replication and transcription machineries moving in opposite orientations (head-on orientation) supposes a considerable risk to genome integrity [2,4,5]. *Escherichia coli* cells have a poor bias for codirectional genes (55% co-directional), although more than 85% of strongly expressed genes were coded in the leading-strand [4]. The analysis of a ColE1 plasmid variant, which initiates unidirectional DNA replication at an R-loop [8], with two replication origins on opposite orientations, revealed that the replication fork initiated at one origin is transiently paused when it encounters a stable R-loop at the other inversely oriented, but topologically silenced, origin [9]. Here, a bubble that spans the distance between the two origins accumulates [9], suggesting that head-on replication–transcription conflicts (RTCs) would compromise genome integrity. The inversion of strongly expressed rRNA (*rrn*) operons to head-on orientation (coded in the lagging-strand) also leads to a transient replication fork stalling [10]. A combination of factors has been proposed to form, resolve or prevent RTCs, including RNA binding and processing factors, DNA helicases, DNA replication- and repair-associated factors, nucleases, etc. In wild-type (*wt*) *E. coli* cells, the Rep DNA helicase (only present in the γ-Proteobacteria Class) in concert with the UvrD or DinG DNA helicase promotes replication across the highly transcribed inverted *rrn* operons and dismantles R-loops [10]. By contrast, in *Bacillus subtilis*, which has a bias for codirectional genes (75% co-directional), strong head-on expression completely blocks DNA replication [11,12,13]. The functions that contribute to genome integrity in response to co-directional RTCs and in the regulation of R-loops are poorly understood in *B. subtilis* cells. It has been proposed that at least PcrA, RecA, and RnhC (counterpart of *E. coli* RnhA) are required for replication across the conflicting region [12,13,14]. (Unless stated otherwise, the indicated genes and products are of *B. subtilis* origin).

PcrA is a UvrD-like DNA helicase that shares a significant degree of structural similarity with *E. coli* UvrD (UvrD*_Eco_*), Rep*_Eco_* and with *Saccharomyces cerevisiae* Srs2 enzyme [15]. PcrA is considered to be an essential enzyme because its deletion or an inactive variant (*pcrA* K37A), whose product lacks ATPase and helicase activities, renders non-viable cells [16,17]. PcrA depletion lethality, however, is suppressed by the *recO16* (formerly termed *recL16*) mutation or by *recA* inactivation, but not by *addAB* (counterpart of *recBCD_Eco_*) inactivation [17,18]. However, the rich-medium synthetic lethality of *E. coli* Δ*uvrD* Δ*rep* cells is not suppressed by *recA* inactivation, but it is fully suppressed by reducing RNAP backtracking or translating ribosomes [19,20,21], suggesting that mitigating RTCs is essential for genomic stability in the Δ*uvrD* Δ*rep* context.

Previously, it has been shown that: (i) RecA forms nucleoprotein filaments on the ssDNA and catalyzes repair-by-recombination, but it can provoke unnecessary recombination [22,23]; (ii) RecA*_Eco_* promotes the annealing between a transcript and the template DNA strand, leading to R-loop formation, via an inverse strand exchange reaction [24,25]; (iii) PcrA, UvrD*_Eco_* or Srs2*_Sce_* limits the loading of the recombinase (RecA*_Eco_* or Rad51*_Sce_*) by dismantling the recombinase nucleoprotein filaments, intermediates crucial for joint molecule formation [26,27,28,29,30]; (iv) PcrA, which physically interacts with the RNAP [31,32], is necessary to mitigate RTCs and to facilitate DNA replication through transcription units [12]; and (v) Rep*_Eco_* in concert with UvrD*_Eco_* or DinG*_Eco_* dismantles, and RnhA*_Eco_* removes R-loops in vivo [10,33]. A likely assumption is that PcrA, by promoting RecA removal from ssDNA, inhibits RecA-mediated R-loop formation and indirectly mitigates RTCs. However, a *Staphylococcus aureus* PcrA (PcrA*_Sau_*) K33A Q250R variant, unable to hydrolyze ATP, can remove RecA from ssDNA [29], but its *B. subtilis* counterpart (PcrA K37A Q254A) is unable to resolve RTCs [12], suggesting that the role of PcrA as an anti-recombinase and on preventing RTCs may be separated functions.

To address how PcrA contributes to remove RTCs, a partial PcrA depletion assay was used in the Δ*rnhB*, Δ*rnhC* or Δ*dinG* context. We show that PcrA depletion lethality is partially suppressed by *rnhB* inactivation, but the lethality is significantly increased in the Δ*rnhC* or Δ*dinG* context. The chromosome segregation defect of PcrA depleted cells is not further increased by *rnhB* or *dinG* inactivation, but chromosome segregation is blocked in the Δ*rnhC* or Δ*recA* context, suggesting that RnhC and RecA might contribute to remove trafficking conflicts. Despite our efforts, we could not construct a Δ*rnhC* Δ*recA* strain, but Δ*rnhB* Δ*recA* and Δ*dinG* Δ*recA* were constructed. Since PcrA depletion lethality partially requires RnhB, we assumed that the toxic intermediates are accumulated due to the absence of the function that removes them rather than because they are formed by PcrA. Using biochemical assays, we have shown that PcrA preferentially binds ssDNA or RNA rather than dsDNA or RNA-DNA hybrids. PcrA catalyzes ssDNA-dependent ATP hydrolysis. PcrA bound to the 3′-tail of duplex DNA unwinds it in the 3′→5′ direction. PcrA preferentially unzips a 3′-tail RNA of a RNA-DNA hybrid over a 3′-tailed duplex. We propose that PcrA in concert with the nucleases RnhC or DinG contributes to process RTCs in order to maintain genetic stability.

## 2. Materials and Methods

### 2.1. Bacterial Strains and Plasmids

All strains were derived from BG214 and its isogenic derivatives are listed in Table 1. The gene to be characterized was deleted by gene replacement, with the *six*-*cat*-*six* (SCS) cassette flanked by homology up and downstream, by a two-step natural chromosomal transformation with selection for an antibiotic resistance [34]. In a second step, the β site-specific recombinase-mediated excision between the two directly oriented *six* sites leads to the deletion of the antibiotic resistance gene and one *six* site, and, as a result, the gene to be characterized is replaced by a single *six* site [35,36]. Accuracy of deletions was confirmed by PCR analysis of the newly constructed strains.

The *pcrA* gene fused to a *ssrA* degradation tag (*pcrA*-*ssrA*) integrated in its native locus and under the control of its native promoter was used to replace the *pcrA* gene, and the *sspB* gene under the control of an isopropyl-β-D thiogalactopyranoside (IPTG)-inducible promoter was ectopically integrated into the *amy* locus to render the degron *pcrA*_T_ strain [12,37]. Upon IPTG addition, the SspB adaptor, expressed from an IPTG-regulated promoter, bound to the SsrA moiety of the PcrA-SsrA fusion protein, and selectively delivers the tagged PcrA-SsrA protein to the ClpXP protease for PcrA degradation (PcrA degron [*pcrA*_T_] strain) [38,39]. The *pcrA*-*ssrA* and *sspB* cassettes of the degron *pcrA*_T_ strain were moved into the Δ*dinG*, Δ*rnhB*, or *rnhC* background by SPP1-mediated generalized transduction as described [17]. The *pcrA*_T_ Δ*recA* strain was also used as a control (Table 1) [17]. The Δ*recO* or Δ*recA* mutations were moved (or tried to move) into the Δ*rnhB*, Δ*rnhC* or Δ*dinG* strains by chromosomal transformation or SPP1-mediated generalized transduction, as described [17,40].

The *pcrA* gene or its Walker A mutant variant *pcrA* K37A were cloned into the pQE1 vector to generate a His-tagged (His_6_-PcrA [pCB1229] and His_6_-PcrA K37A [pCB1230]) variant (Table 1).

### 2.2. Viability and Survival Assays

PcrA degron cultures were grown to OD_560_ = 0.4 with shaking at 37 °C, and with a doubling time of 29–33 min. The cultures were divided and aliquots plated in LB agar plates with or without 500 μM IPTG (Calbiochem, Madrid, Spain). Colony forming units (CFUs) in LB agar plates containing or not IPTG were measured. The mean and the standard error of mean (SEM) were calculated using the R software (The R Foundation, Vienna, Austria), and a Student’s *t*-test was performed to denote the threshold of significance.

**Figure 1:**
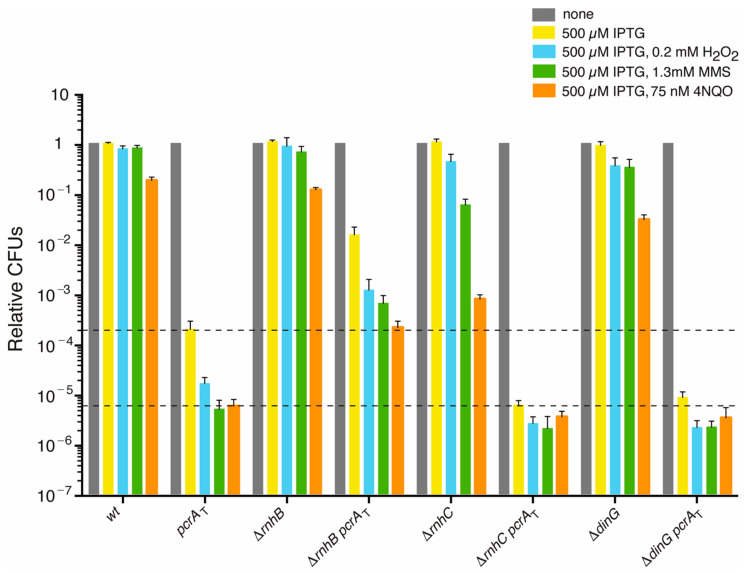
PcrA lethality is partially suppressed by *rnhB* inactivation, but not by *rnhC* or *dinG* inactivation. Log phase cultures of *wt*, single (*pcrA*_T_, Δ*rnhB*, Δ*rnhC* or Δ*dinG*) and double mutant (*pcrA*_T_ Δ*rnhB*, *pcrA*_T_ Δ*rnhC* or *pcrA*_T_ Δ*dinG*) strains were diluted, plated on LB agar and incubated overnight at 37 °C (grey bars). Lethality assays showing cell viability upon PcrA depletion in the *pcrA*_T_, Δ*rnhB pcrA*_T_, Δ*rnhC pcrA*_T_, or Δ*dinG pcrA*_T_ strain containing 500 μM IPTG (yellow bars). Log phase cultures of the indicated strains were diluted and plated on LB agar containing IPTG (500 µM, yellow bars) and H_2_O_2_ (0.2 mM, blue bars), MMS (1.3 mM, green bars) or 4NQO (75 nM, orange bars). Experiments were performed at least four times. The dotted lines mark the upper and lower limit of the cell viability rate upon PcrA depletion. Data are shown as the mean fractional survival ± SEM.

Methyl methanesulfonate (MMS), H_2_O_2_ or 4-nitroquinoline-1-oxide (4NQO) were from Sigma Aldrich (Merck KGaA, Darmstadt, Germany). Cell sensitivity to chronic H_2_O_2_, MMS or 4NQO exposure was determined by growing cultures to OD_560_ = 0.4 and plating appropriated dilutions on LB agar plates supplemented with the indicated concentrations of H_2_O_2_ (0.2 mM), MMS (1.3 mM) or 4NQO (75 nM) and the presence or absence of IPTG (500 μM) as described [17]. Plates were incubated overnight (16–18 h, 37 °C) and the number of CFUs determined (Figure 1). Experiments were conducted independently at least four times. Fractional survival data are shown as mean ± SEM. Statistical analysis was performed with a two-tailed Student’s *t*-test. For experiments involving more than two groups, one-way analysis of variance (ANOVA) was performed. For all tests, a *p*-value of <0.05 was considered as significant and a *p*-value > 0.1 was considering as non-significant. All statistical analyses were performed using the R software.

### 2.3. Fluorescence Microscopy and Data Analysis

For chromosome segregation analyses, cells were fixed and stained as described [41]. To obtain exponentially growing cells, overnight cultures were inoculated in LB rich medium. Cells were grown unperturbed in LB medium to OD_560_ = 0.2 with shaking at 37 °C. IPTG (500 μM) was added to half of the culture, and both cultures were further incubated (60 min, 37 °C). Then, cells were collected, subjected to fixation with 2% formaldehyde, and finally stained with 4′,6′-diamino-2-phenylindole (DAPI) staining (1 μg/mL). Samples were visualized and photographed by fluorescence microscopy with a Hamamatsu 3CCD Digital Camera C7780 (Hamamatsu, Japan) coupled to a BX61 Olympus fluorescence microscope (Tokyo, Japan), equipped with a 100× immersion oil lens and a DAPI filter (U-MNU2).

The ImageJ software (NIH, Bethesda, MD, USA) was used to merge the phase contrast and DAPI-fluorescence images, which allowed us to distinguish the septum, and thus determine the filamentation event, and was also used to determine the cell length. Blind scoring was performed on captured images as described [41].

### 2.4. Enzymes, Reagents, Protein, and DNA Purification

All chemicals used were analytical grade. DNA restriction enzymes and DNA ligase were from New England Biolabs (Ipswich, MA, USA), and polyethyleneimine, DTT, ATP, and dATP were from Sigma-Aldrich (Merck KGaA, Darmstadt, Germany). DEAE Sepharose and His-Trap HP were from GE Healthcare (Chicago, IL, USA), and phosphocellulose was from Whatman (Maidstone, UK).

PcrA and its mutant variant PcrA K37A (Walker A motif mutant K37A) were found to be prone to proteolytic degradation during purification, thus both proteins were purified with a His-tag, which was removed upon protein purification. Proteins were purified from *E. coli* M15 (pREP4) cells transformed with His_6_-PcrA (pCB1229) and His_6_-PcrA K37A (pCB1230) (Table 1). In short, cells were grown at 30 °C in LB medium to *A*_600_ ~0.5 and then 1 mM IPTG was added to induce protein expression. Cultures were further incubated (3 h at 30 °C), and then cells were harvested by centrifugation (6000× *g*, 15 min at 4 °C). The cell pellet (~3 g/L of wet cell mass) was resuspended in buffer A (50 mM Tris-HCl pH 7.5, 1 M NaCl, 0.5% Brij-58, 50 mM imidazole, 1 mM *p*-NH_2_-benzamidine, 20% glycerol). A tablet of EDTA-free protease inhibitors cocktail (Roche, Basel, Switzerland) was added, and cells were disrupted using a French Press in an ice bath. Cell debris was separated from the soluble lysates by centrifugation (18,000× *g*, 15 min at 4 °C).

The soluble PcrA extract was loaded into a Ni^2+^-activated His-Trap chelating 5 mL column set-up pre-equilibrated with 10 volumes of buffer B (50 mM Tris-HCl pH 7.5, 0.5 M NaCl, 10% glycerol) containing 50 mM imidazole using an ÅKTA purifier (GE Healthcare, Chicago, IL, USA). After washing the column with 10 volumes of buffer B containing 50 mM imidazole, the protein was eluted in a one-step elution with buffer B containing 400 mM imidazole. Fractions of His-PcrA or His-PcrA K37A (0.2 mL) were collected and analyzed by SDS–PAGE. The fractions containing the His-PcrA or His-PcrA K37A protein were pooled. The His-tag was removed upon protein purification using the TAGZyme system (Qiagen, Hilden, Germany). Protein buffer was changed to store buffer C (50 mM Tris-HCl pH 7.5, 300 mM NaCl, 0.01% Triton X-100, 1 mM DTT) containing 50% glycerol by extensive dialysis at 4 °C. Proteins were finally snap frozen and stored at −80 °C.

The corresponding molar extinction coefficient for PcrA was calculated as 70,375 M^−1^ cm^−1^ at 280 nm, as previously described [42]. The protein concentrations were determined using the above molar extinction coefficient. PcrA and its variant are expressed as moles of monomers.

### 2.5. DNA Binding Assays

The nucleotide sequence of the oligonucleotides used are indicated in the 5′→3′polarity: 170, AGACGCTGCCGAATTCTGGCTTGGATCTGATGCTGTCTAGAGGCCTCC- ACTATGAAATCG; 171, CGATTTCATAGTGGAGGCCTCTAGACAGCA; 173, AGCTC- ATAGATCGATAGTCTCTAGACAGCATCAGATCCAAGCCAGAATTCGGCAGCGTC T; 172, TGCTGTCTAGAGACTATCGATCTATGAGCT; Fork1, CGGCATTCGTGCCAAG CTTGCATGCCTGCAGGTCGACTCTAGAGGATCCCCGGGTACCGAGCTCGAATTC ACTGGCCGTCGTTTTACAACGTCGTGACTGG; 345, GCGATTTCATAGTGGAGGCC TCTAGACAGCACGCCGTTGAATGGGCGGATGCTAATTACTATCTC; 346, GAGATA GTAATTAGCATCCGCCCATTCAACGGCGTGCTGTCTAGAGACTATCGATCTATG AGCTCTGCAGC; SM44R, GCUCUGAUGCCGCAUAGUUAAGCCAGCCCCGACACC CG; SM44D, GCTCTGATGCCGCATAGTTAAGCCAGCCCCGACACCCG; SM45D, CG GGTGTCGGGGCTGGCTTAACTATGCGGCATCAGAGC; SM46D, GCTCTGATGCCGC ATAGTTAAGCCAGCCCC; SM47D, CGGGTGTCGGGGCTGGCTTAACTATGCGGC. As revealed in Supplementary Figure S1, the different DNA substrates were assembled by annealing the indicated oligonucleotides. The ssDNA concentrations were measured using the extinction coefficient of 1.54 × 10^−4^ M^−1^ cm^−1^ at 260 nm, and the concentrations of DNA were expressed as moles of DNA molecules. The annealed products (dsDNA, flayed, 3′- or 5′-fork DNA, 3′- or 5′-tailed dsDNA, etc.) were gel purified as described and stored a 4 °C [42,43].

DNA binding was assayed by electrophoretic mobility shift assays (EMSAs) using the [γ^32^P]-labeled DNAs (0.1 nM) in buffer D (20 mM Tris-HCl pH 7.5, 50 mM NaCl, 3 mM MgCl_2_, 4 mM DTT, 0.05 mg/mL BSA, 5% glycerol) containing 2.5 mM ATPγS. Reactions were incubated for 15 min at 37 °C. Protein-DNA complexes were separated using 5% (*w*/*v*) polyacrylamide gel electrophoresis (PAGE) in 0.25× Tris-borate EDTA (TBE) buffer, and visualized by autoradiography.

### 2.6. Nucleotide Hydrolysis Assays

The ssDNA-dependent (d)ATP hydrolysis activity of PcrA and its variant (PcrAK37) was assayed via a NAD/NADH coupled spectrophotometric enzyme assay [44]. Reactions were incubated in buffer E (50 mM Tris-HCl [pH 7.5], 80 mM NaCl, 10 mM MgOAc, 50 μg·mL^−1^ BSA, 1 mM DTT, 5% glycerol) containing 5 mM (d)ATP [44] and an ATP regeneration system (300 μM NADH, 100 U/mL of lactate dehydrogenase, 500 U/mL pyruvate kinase, and 2.5 mM phosphoenolpyruvate), in a final volume of 50 μL (30 min, 37 °C). The order of addition of 3199-nt pGEM3 Zf(+) ssDNA, poly dT or SM44R RNA (10 µM in nt) and purified PcrA are indicated in the text. Data obtained from (d)ATP hydrolysis were converted to ([d]ADP) and plotted as a function of time [44]. The ATPase activity was determined monitoring the disappearance of absorbance at 340 nm, due to NADH conversion to NAD, using a Shimadzu CPS-20A (Tokyo, Japan) dual-beam spectrophotometer. Reactions were repeated at least three times, and the rate of PcrA-mediated (d)ATP hydrolysis (K_cat_), derived from the slope of each curve is shown as the mean ± standard deviation (SD). The lag time, which represents the delay in reaction progress relative to a theoretical reaction curve that lacks a lag time, was derived from the time intercept of a linear regression line fit to the steady state portion of data [44]. A standard curve with known amounts of NADH was obtained and used to convert the drop-in absorbance/time to (d)ADP concentration/time [44]. V_max_ and K_m_ values were calculated by constructing Michaelis–Menten plots using the R software as described [45].

### 2.7. DNA Unwinding Assays

The DNA substrates were incubated in buffer D containing 2.5 mM ATP with increasing PcrA (or PcrA K37A) concentrations (15 min, 37 °C), or with a fixed PcrA concentration for a variable time at 37 °C in a final volume of 20 μL, as previously described [46]. The reaction was stopped by adding 5 μL of stopping solution (50 mM EDTA, 0.5 μg proteinase K, 0.5% (*w*/*v*) SDS in DNA loading buffer) (5 min, 37 °C). The substrates and products were separated using a 6% PAGE in 1× Tris-glycine (TG) or a 15% PAGE in 0.5× TBE buffer. Gels were run and dried prior to autoradiography. The substrate and resulting products were quantified as described [47].

## 3. Results

### 3.1. Experimental Rationale

In *E. coli* cells, there are two (Rep and UvrD), and, in Firmicutes, there is one (PcrA) UvrD-like DNA helicase, and they move in a 3′→5′ direction [15]. PcrA expression complements Δ*uvrD_Eco_*, but not Δ*rep_Eco_* defects. Furthermore, PcrA expression inactivates the function of Rep, providing a heterologous dominant negative phenotype [16]. The phenotypes associated with PcrA depletion are complex and reflect its involvement in several DNA transactions. PcrA (as UvrD*_Eco_*) contributes to: (i) the removal of bulky lesions by nucleotide excision repair (NER), with UvrA acting as a DNA damage sensor and PcrA (UvrD*_Eco_*) acting after damage incision via the global-genome NER sub-pathway [48,49]; (ii) by interacting with the RNAP [31,32], which acts as a DNA damage sensor on the transcribed strand, backtrack RNAP to expose lesions; and also interacts with UvrB to load the UvrBC complex to remove the lesions via a minor transcription coupled DNA-repair (TCR) sub-pathway [48,49]; and (iii) the removal of replication impairments as protein roadblocks (e.g., RecA assembled on ssDNA) or helping to mitigate RTCs [12]. Currently, the role of PcrA and which other function(s) contribute(s) to PcrA inviability are poorly understood.

In *E. coli*, Rep in concert with UvrD or DinG appears to promote replication across highly transcribed regions and to have a role in removing R-loops in vivo [10]. Here*,* DinG*_Eco_*, which moves in a 5′→3′ direction, unwinds a number of unusual RNA-DNA hybrid structures [50,51]. *E. coli* cells encode for two XPD-like enzymes (DinG and YoaA). In Firmicutes, there is only one XPD-like enzyme, whose expression is not damage inducible [52], although it is still termed *dinG*. DinG lacks the N-terminal cysteine residues, required to form the Fe-S cluster essential for helicase activity [51]. DinG has an N-terminal exonuclease-like domain absent in DinG*_Eco_* and YoaA*_Eco_* [53]. DinG*_Sau_* has a 3′→5′-exonuclease activity, and with significant efficiency degrades RNA of RNA-DNA hybrid substrates, but lacks DNA helicase activity [53]. The helicase activities of DinG*_Eco_* and the R-loop unwinding of RecG*_Eco_* are not observed in their *B. subtilis* counterpart enzymes [53,54]. The contribution of DinG, which shares 25% identity with DinG*_Eco_* (between residues 238 and 916), on PcrA depletion lethality is unknown.

The persistence of R-loops is an endogenous source of RTCs, and the RNase H family of enzymes recognizes and degrades the RNA moiety of the hybrid RNA-DNA structures [3,4,5]. Phylogenetic studies have shown that all prokaryotic genomes analyzed contain at least one RNase H enzyme, with most genomes containing RNase HI and HII, and a subset containing RNase HII and RNase HIII [55]. *E. coli* cells have two RNases H enzymes (RnhA [RNase HI] and RnhB [RNases HII]), and *B. subtilis* has two functional RNase H enzymes (RnhB [RNase HII] and RnhC [RNase HIII]) [55,56,57,58]. Only in few cases is the coexistence of functional RNase HI and HIII in the same genome documented. *Mycobacterium smegmatis* has four genuine RNase H-like enzymes (RnhA, RnhB, RnhC and RnhD), but many genera of the *Actinobacteria* Phylum contain RnhC, and are devoid of RnhA (e.g., *M. tuberculosis*) [59].

The replisome can bypass a rNMP mis-insertion, at a cost of delaying its progression by 4 to 30-fold [60]. However, an rNMP mis-insertion stalls the elongation complex and RNAP must be backtracked and the rNMP(s) removed [4,61]. The primary function of RnhB is to remove mis-incorporated rNMPs via the ribonucleotide excision repair (RER) pathway [62]. RnhB recognizes and nicks the ribose sugar in duplex DNA to remove a single or very few mis-incorporated rNMPs residues (<4-nt), and then YpcP (also termed ExoR) and DNAP I prepare the substrate for ligation [57,63]. When RER is impaired, the global-genome nucleotide excision repair (NER) and minor TCR sub-pathways, by recognizing structural and conformational changes on DNA induced by mis-incorporated rNMPs, remove them [64]. RnhC, which is physically associated with RNAP even in the absence of exogenous DNA damage [31], recognizes and cleaves the RNA portions of the R-loops at replication initiation sites, arising during RTCs, and the mis-maturated Okazaki primers [13,57]. The activities associated with RnhB and RnhC on PcrA depleted cells are unknown.

To test the contribution of PcrA on RTCs, the *pcrA* tagged (*pcrA*_T_) degron cassettes (*pcrA*-*ssrA* and *sspB*) were moved into the null *rnhB* (Δ*rnhB*), Δ*rnhC* or Δ*dinG* strain (Table 1), and cells were plated on agar plates containing IPTG or IPTG and a cytotoxic agent. After IPTG addition, the expression of the SspB adaptor, which marks PcrA-SsrA for degradation by the ClpXP protease, is induced (see Material and methods). Indeed, within the first 15 min of 100 μM IPTG addition, the PcrA level is reduced by 60–90% in the *pcrA*_T_ strain, and cell viability decreases >1000-fold (*p* < 0.01) (Figure 1) [12].

Discordance between the replication and transcription machinery as they move along a common DNA template, and a genotoxic stress increases genomic instability. Thus, the Δ*rnhB pcrA*_T_, Δ*rnhC pcrA*_T_, or Δ*dinG pcrA*_T_ cells were exposed to IPTG and limiting MMS, H_2_O_2_, or 4NQO concentrations. MMS is an alkylating agent that induces damaged template bases, H_2_O_2_ induces oxidative damages on template bases and single-stranded nicks, and the UV mimetic 4NQO induces bulky adducts on purines. The H_2_O_2_-induced damaged template bases are removed from duplex DNA by base excision repair (BER), and the single-stranded nicks, which can be converted into one-ended double-strand breaks (DSB) when the replisome collides with them, are repaired by HR [65,66]. The MMS-induced damaged template bases are specifically removed from duplex DNA by BER or direct reversal [66,67]. On the other hand, the 4NQO-induced bulky lesions on template strands are specifically removed from duplex DNA by global-genome NER and by the minor TCR sub-pathways [48,49]. If the H_2_O_2_-, MMS- or 4NQO-induced lesions escape their specialized repair pathways (e.g., if they are in ssDNA), HR functions have to circumvent or bypass the lesion or to contribute to DSB repair [66].

### 3.2. PcrA Lethality Is Partially Suppressed by rnhB Inactivation

To understand the cause or consequence of the lethality upon PcrA depletion, we have induced a replication stress by the accumulation of rNMP mis-insertions under conditions in which PcrA can be depleted by using the *pcrA*-*ssrA sspB* Δ*rnhB* strain (for simplicity *pcrA*_T_ Δ*rnhB*) (Table 1), and cell viability was analyzed (Figure 1).

The *wt*, *pcrA*_T_, Δ*rnhB*, or Δ*rnhB pcrA*_T_ strains were grown in rich LB medium to an OD_560_ = 0.4 (~5 × 10^7^ CFUs/mL) at 37 °C with shaking. In the absence of IPTG, the *pcrA*_T_, Δ*rnhB*, or Δ*rnhB pcrA*_T_ strain shows no defect in plating efficiency on LB agar plates when compared to the *wt* control strain (Figure 1 and Figure S2, grey bars; *p* > 0.05). In the presence of 500 μM IPTG, however, PcrA depletion lethality was partially suppressed by *rnhB* inactivation (Figure 1, yellow bars). Here, the viability of the Δ*rnhB pcrA*_T_ strain was significantly increased (by ~77-fold; *p* < 0.01) when compared to the *pcrA*_T_ control strain (Figure 1, yellow bars). The suppression was only partial because the plating efficiency of the Δ*rnhB pcr*A_T_ strain was reduced by ~65-fold (*p* < 0.01) when compared to the absence of IPTG (Figure 1, yellow vs. grey bar). Since the viability of the *pcrA*_T_ strain is not further decreased by increasing the IPTG concentrations, and the presence of 500 μM IPTG neither affects cell viability of the *pcrA*-*ssrA* or *sspB* intermediate strains [17] nor of the Δ*rnhB* strain, it is likely that lethality is a direct consequence of the partial PcrA depletion. It remains unknown the mechanism of partial suppression upon IPTG addition on the Δ*rnhB pcrA*_T_ strain.

We can envision that: (i) PcrA inviability might also require conditions that cannot compensate for its activity, but, for the avoidance of RTCs; and (ii) rNMP mis-insertions, which slow down replication and halt transcription elongation [4,61], provide more time for alternative pathways to remove the toxic intermediates accumulated upon PcrA depletion. This hypothesis is consistent with the observation that rich medium synthetic lethality of *E. coli* Δ*uvrD* Δ*rep* cells is fully suppressed by mutations that compromise RNAP backtracking or translation elongation, and indirectly alleviate RTCs [19,21].

### 3.3. PcrA Is Required to Overcome a Replicative Stress

In *E. coli* and *S. cerevisiae*, RTCs and backtracked RNAP elongation complexes often result in DSBs that are differentially repaired in the presence or absence of RNase H enzymes [7,68]. To analyze whether H_2_O_2_-induced single-stranded nicks significantly increase the collapse of DNAP or RNAP and if a genotoxic stress differentially affects PcrA depletion lethality in the Δ*rnhB* context, the Δ*rnhB pcrA*_T_ strain was exposed to limiting concentrations of H_2_O_2_, MMS, or 4NQO (see Section 3.1).

The *wt*, *pcrA*_T_, Δ*rnhB*, and Δ*rnhB pcrA*_T_ strains were grown in LB medium to an OD_560_ = 0.4 (~5 × 10^7^ CFUs/mL) with shaking, at 37 °C. The plating efficiency of Δ*rnhB* cells grown in LB agar plates under unperturbed conditions was similar to that of the *wt* control (Figure 1 and Figure S2, grey bars; *p* > 0.1), suggesting that the presence of rNMP mis-insertions does not significantly compromise the plating efficiency. The survival of the single Δ*rnhB* mutant strain was not significantly affected (*p* > 0.1) by plating on 0.2 mM H_2_O_2_ or 1.3 mM MMS containing plates, and was moderately decreased (by ~3-fold; *p* < 0.05) on plates containing 75 nM 4NQO when compared to the *wt* control, both in the absence or presence of 500 μM IPTG (Figure 1 and Figure S2). IPTG, however, was included in the analysis of the single mutant strains to have only one experimental variable (the clastogen) in the reactions with the double mutant strains (Figure 1, blue, green, and orange vs. a grey bar).

The survival of the Δ*rnhB pcrA*_T_ strain was not significantly affected (*p* > 0.1) by plating on 0.2 mM H_2_O_2_ or 1.3 mM MMS containing plates, and was decreased (~3-fold; *p* < 0.05) on plates containing 75 nM 4NQO when compared to the most sensitive (Δ*rnhB*) single mutant strain (Figure S2, blue, green, and orange bars). In other words, the survival of the double mutant strain to 4NQO was decreased by ~10-fold (*p* < 0.01) when compared to the *wt* control strain (Figure S2, orange vs. grey bars), suggesting that the PcrA-SsrA fused protein and/or a noise of the *sspB* gene expression and the presence of rNMP mis-insertions or lack of cleavage of the rNMPs by *rnhB* inactivation marginally impairs cell survival of the Δ*rnhB pcrA*_T_ strain.

Partial PcrA depletion, by IPTG addition, and exposure to H_2_O_2_, MMS or 4NQO significantly reduced *pcrA*_T_ survival by ~12-, ~38- and ~33-fold, respectively, when compared to the only-IPTG condition (Figure 1, blue, green, and orange vs. yellow bar; *p* < 0.01). Thus, PcrA contributes to cell survival upon exposure to H_2_O_2_, MMS, or 4NQO. Similar results were observed upon exposure of *pcrA*_T_ cells to H_2_O_2_ or MMS [17]. Unfortunately, acute exposure to IPTG and H_2_O_2_, MMS, or 4NQO for min 15 min, and then plating cells on LB agar plates lacking both IPTG and H_2_O_2_, MMS or 4NQO is not informative [17]. This is consistent with the observation that upon DNA damage, cells require up to 180 min for replication re-start [69], but in <15 min PcrA should reach *wt* levels in the absence of IPTG (see [12]).

To test whether PcrA and RnhB work in concert to remove H_2_O_2_-, MMS-, and 4NQO-induced lesions, the Δ*rnhB pcrA*_T_ cells were plated on LB agar plates containing IPTG and limiting concentrations of H_2_O_2_, MMS, or 4NQO. The survival of the Δ*rnhB pcrA*_T_ strain was significantly decreased by ~12-, ~23-, and ~70-fold, respectively, when compared to the only-IPTG condition (Figure 1, blue, green, and orange vs. yellow bar; *p* < 0.01). However, the survival of Δ*rnhB pcrA*_T_ cells was increased by ~7 and ~4-fold (*p* < 0.05) in the presence of IPTG and H_2_O_2_ or MMS, respectively, whereas it was not significantly increased (by ~1.1-fold; *p* > 0.1) in the presence of IPTG and 4NQO when compared to the *pcrA*_T_ strain in the absence of their respective clastogens (Figure 1, blue, green, and orange vs. yellow bar).

These data altogether suggest that: (i) *rnhB* inactivation does not contribute to the removal of H_2_O_2_-induced oxidative damage (including 8-oxoguanine), which delays but only marginally arrests DNAP and RNAP progression [70]; (ii) upon PcrA depletion, the H_2_O_2_-induced damaged template bases and single-strand nicks, which through replication are converted in one-ended DSBs, enhance cell survival in the Δ*rnhB* context; (iii) *rnhB* inactivation does not contribute to the removal of MMS-induced DNA lesions, the MMS-induced damaged template bases (including O^6^-methylguanine, O^4^-methylthymine), which block both DNAP and RNAP progression [67], are repaired by alternative pathways, and *rnhB* inactivation partially suppresses cell survival of MMS lesions on PcrA depleted cells; (iv) the rNMP mis-insertion sensitizes Δ*rnhB* cells to 4NQO-induced lesions, and the Δ*rnhB pcrA*_T_ strain is deficient in the removal of bulky lesions when compared to the *wt* strain; and (v) *rnhB* inactivation does not significantly suppresses cell survival of 4NQO-induced lesions on the PcrA depleted condition, suggesting that mis-incorporated rNMPs might titrate functions involved in global-genome NER.

### 3.4. PcrA Inviability Does Not Require RnhC or DinG

We can envision that PcrA inviability might result from the accumulation of RTCs in the Δ*rnhC* or Δ*dinG* context (see Section 3.1). To test the hypothesis, the *pcrA_T_* cassettes were moved into the Δ*rnhC* or Δ*dinG* mutant strain via SPP1-mediated generalized transduction (Table 1). The Δ*rnhC pcrA*_T_ or Δ*dinG pcr*A_T_ cells were grown in an LB medium to an OD_560_ = 0.4 with shaking, at 37 °C. Then, appropriate dilutions were plated on LB agar plates containing 500 μM IPTG. The lethality upon PcrA depletion was not suppressed by *rnhC* or *dinG* inactivation (Figure 1, yellow bars). On the contrary, the plating efficiency of the Δ*rnhC pcr*A_T_ or Δ*dinG pcr*A_T_ strain was significantly reduced (by ~32- and ~23-fold, respectively; *p* < 0.01), when compared to the *pcrA*_T_ control in IPTG containing plates (Figure 1, yellow bars). This suggests that an endogenous source of genome instability (e.g., RTCs) significantly decreases the viability of the Δ*rnhC pcr*A_T_ or Δ*dinG pcr*A_T_ strain in the presence of IPTG. In other words, the plating efficiency of the Δ*rnhC pcr*A_T_ or Δ*dinG pcr*A_T_ strain was decreased by >1 × 10^5^-fold (Figure 1, yellow vs. grey bar; *p* < 0.01), suggesting that, upon PcrA depletion, the absence of RnhC or DinG may cause trafficking conflicts, and increases cell death. In *E. coli*, however, Δ*uvrD* and Δ*rnhA* mutations are synthetically lethal, but the Δ*uvrD* and Δ*dinG* mutations are not [10,71].

The plating efficiency of Δ*rnhC* or Δ*dinG* cells grown in LB medium under unperturbed conditions was similar to that of the *wt* control (Figure 1 and Figure S2, grey bars; *p* > 0.1). In the absence or presence of IPTG, the survival of the single Δ*rnhC* mutant strain was not significantly affected upon exposure to H_2_O_2_ (*p* > 0.1), but it was strongly reduced (by ~14- and ~200-fold; *p* < 0.05 and *p* < 0.01, respectively) upon exposure to MMS or 4NQO, respectively, when compared to the *wt* control (Figure 1 and Figure S2, green and orange bars). The survival of the Δ*rnhC* single mutant strain to IPTG and 4NQO was strongly reduced (by ~750-fold; *p* < 0.01) when compared to the minus 4NQO condition (Figure 1 and Figure S2, orange vs. grey bar). Unlike Δ*rnhC* cells (Figure 1 and Figure S2), Δ*rnhC_Msm_* cells remain recombination proficient and are as capable of repairing non-bulky oxidative damage and bulky UV-induced lesions as the *wt* control [59]. Here, RnhA_Msm_ contributes to remove the RNA-DNA hybrids [59], but *B. subtilis* lacks a functional RnhA enzyme using conventional substrates (see Section 3.1).

The survival rate of the single Δ*dinG* mutant strain was marginally reduced (by ~2 and ~2.5-fold; *p* > 0.1) upon exposure to IPTG and H_2_O_2_ or MMS, respectively, and it was reduced (by ~7-fold; *p* < 0.05) upon exposure to 4NQO when compared to the *wt* control, both in the presence or absence of IPTG (Figure 1 and Figure S2, blue, green, and orange bars).

Upon PcrA depletion, the total number of viable cells was extremely reduced in the Δ*rnhC pcr*A_T_ or Δ*dinG pcr*A_T_ context, thus we doubted whether the cellular responses to different clastogens might provide reliable results with those small limits of detection. Anyhow, we have tested whether PcrA and RnhC or DinG work in concert to remove H_2_O_2_-, MMS-, and 4NQO-induced lesions. Upon addition of IPTG and limiting H_2_O_2_, MMS or 4NQO, the survival of the Δ*rnhC pcr*A_T_ or Δ*dinG pcr*A_T_ strain was marginally decreased (between 1.5- to 4-fold) when compared to the absence of DNA damage (Figure 1, blue, green, and orange vs. yellow bar). We wonder whether these low differences in response to IPTG and limiting H_2_O_2_-, MMS- or 4NQO-induced DNA damage are genuine or are due to a high operational error from our assays. Indeed, from exponentially growing Δ*rnhC pcrA*_T_ or Δ*dinG pcr*A_T_ cells (~5 × 10^7^ CFUs/mL), we have to plate 1 mL of the cell culture on plates containing IPTG and limiting H_2_O_2_, MMS, or 4NQO concentrations to count between 50 to 90 CFUs/plate. Alternatively, cell persistence could distort our analysis. Persistence, which is the non-inheritable ability of a small subpopulation of poorly or non-growing cells to survive exposure to an otherwise lethal concentration of a clastogen, might account for the low significant difference in our cell survival assays. To test whether PcrA works in concert with RnhC or DinG, we have performed chromosomal segregation studies.

### 3.5. PcrA Depletion Leads to Unsegregated Chromosomes in the ΔrnhC Context

**Figure 2:**
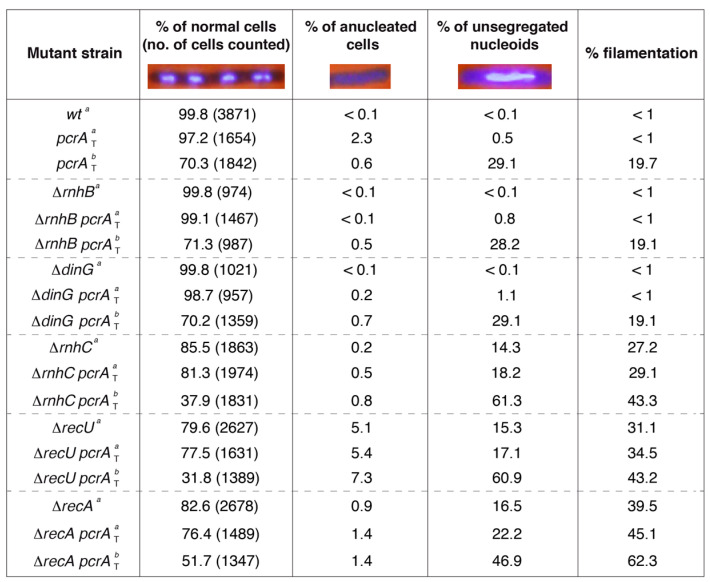
PcrA depletion leads to unsegregated chromosomes in the Δ*rnhC* and Δ*recA* context. The indicated strains were grown in LB medium to OD_560_ = 0.2 (37 °C), the culture was split and 500 μM IPTG was added (condition b) or not (condition a). After 60 min, the cultures were harvested, prepared for DAPI DNA-fluorescence microscopy, and the percentage of normal, anucleate, and unsegregated nucleoids determined. The fraction of filamented cells is indicated. Representative fluorescent images of two dividing DAPI-treated cells (DNA stain, light blue) are shown. The pictures are taken at the same amplification. Two not separated cells (four nucleoids) are presented under normal conditions in ~60% of total dividing cells. The mean ± SEM of at least three independent experiments is shown.

Branched recombination intermediates are formed at RTCs, and behind a stalled replication fork the daughter strands are intertwined, forming precatenanes [3,4]. The cells with linked daughter strands or unresolved branched intermediates fail to separate their nucleoids, and this is accompanied by a delay in chromosomal segregation. The absence of the RecU Holliday resolvase, which plays a crucial role in the resolution of the branched intermediates, causes the accumulation of unresolved chromosomes (anucleated cells) and a severe chromosomal segregation defect [41,72]. PcrA depletion provokes a chromosomal segregation defect [17]. The chromosomal segregation defect of Δ*rnhB pcrA*_T_, Δ*rnhC pcrA*_T_ or Δ*dinG pcrA*_T_ cells was analyzed; and, as control, the Δ*recA pcrA*_T_ and the Δ*recU pcrA*_T_ strains were also studied (Figure 2). The plating efficiency of the Δ*recA* or Δ*recU* strain is reduced during unperturbed growth at 37 °C in LB medium. Only ~20% of total ∆*recA* or ∆*recU* cells could form colonies on plates [41].

The single and double mutant strains were grown in LB medium under unperturbed conditions until they reached OD_560_ = 0.2 with shaking (at 37 °C). IPTG (500 μM) was added to half of the culture to induce PcrA depletion, and both cultures were further incubated (60 min, 37 °C). Then, cells were harvested, fixed, and stained with DAPI and analyzed by fluorescence microscopy as described (Materials and Methods). During vegetative growth, cells are 2–4 μm long, and net accumulation of anucleated and unsegregated chromosomes was rare in Δ*rnhB*, Δ*dinG,* or *wt* cells in the absence or presence of IPTG (Figure 2). In ~14% of Δ*rnhC* cells, a chromosomal segregation defect was observed, and ~27% of total cells were elongated when compared to the *wt* control (Figure 2). Those elongated cells (>6 μm in length) contained a single nucleoid and, after the merge of the phase contrast and DAPI-fluorescence images, no septum could be inferred [17], suggesting that, in the absence of RnhC, the accumulated RTCs lead to a chromosomal segregation defect and to filamented cells. Similar defects were observed when the Δ*recA* or Δ*recU* single-mutant strains were analyzed, but here the proportion of anucleated (absence of DAPI) was increased by ~10- and ~50-fold, respectively (Figure 2) [41].

In the presence of IPTG for 60 min and subsequent PcrA depletion, the proportion of anucleated, cells with aberrant chromosomes and filamented cells was not significantly increased in the Δ*dinG* and Δ*rnhB* context when compared with the parental *pcrA*_T_ strain (*p* > 0.1) (Figure 2). By contrast, upon PcrA depletion, the proportion of unsegregated nucleoids and filamented cells was significantly increased (by >600- and >40-fold, respectively, *p* < 0.01) in the Δ*rnhC* strain, when compared to the *wt* control (Figure 2). The total number of unsegregated cells accounted to ~60% in the Δ*rnhC pcrA*_T_ context when compared to the *pcrA*_T_ control (~30%) (Figure 2). In the Δ*rnhC pcrA*_T_ or Δ*recA pcrA*_T_ cells, the proportion of anucleated cells marginally increased (by ~1.3- and ~2.3-fold, *p* > 0.1), whereas, in the Δ*recU pcrA*_T_, the proportion of anucleated cells was increased (by ~12-fold, *p* < 0.01) when compared to the *pcrA*_T_ strain, but was marginally increased (by ~1.3-fold, *p* > 0.1) when compared to the Δ*recU* strain (Figure 2) [41].

These data altogether suggested that: (i) a Δ*rnhB* or Δ*dinG* mutation does not contribute to mitigate the chromosomal segregation defect observed upon PcrA depletion; (ii) unresolved branched intermediates accumulate in cells lacking RnhC, and this defect is significantly aggravated by PcrA depletion in the Δ*rnhC* context; and (iii) upon PcrA depletion, accumulation of branched intermediates and R-loops causes a chromosomal segregation defect and such defect was increased in the Δ*rnhC* context, but not in the Δ*dinG* background. It is likely, therefore, that RnhC and DinG contribute differentially to the processing of branched intermediates, but in cells depleted of PcrA, the absence of RnhC or DinG strongly compromises cell viability in the absence of exogenous DNA damage.

### 3.6. The ΔrecA Mutation Is Synthetically Lethal in the ΔrnhC Context

In vitro, the recombinases from *E. coli* and *S. cerevisiae* origin, RecA and Rad51, promote annealing between a transcript and the template DNA strand, leading to R-loop formation, via an inverse strand exchange reaction [24,25,73], and the R-loops are removed by RNase HI to restore growth [4,33]. Paradoxically, PcrA depletion lethality is suppressed by *recA* inactivation [17], but cell viability is significantly decreased by *rnhC* inactivation (Figure 1). Furthermore, results from the previous section suggested that RecA and RnhC are vitally important to process branched intermediates that result by PcrA depletion. To test whether redundant mechanisms ameliorate the RTC defect observed upon inactivation of the RNA processing activities of RnhC, we have tried to move the Δ*recA* mutation into the Δ*rnhC* background by SPP1-mediated transduction. As a control, we have moved the Δ*recA* mutation into the Δ*rnhB* strain.

We have constructed the Δ*recA* Δ*rnhB* (BG1753) strain (Table 1), which showed no apparent growth defect under unperturbed growth conditions when compared to the Δ*recA* strain. However, despite our efforts, we could not mobilize the Δ*recA* mutation in the Δ*rnhC* context. Similar results were obtained when we tried to move the Δ*recA* mutation into the Δ*rnhC* background by natural chromosomal transformation. From the data reported in Figure 1, it could be assumed that DinG, which is a single-stranded exo(ribo)nuclease [53], contributes to cell viability upon PcrA depletion. We have successfully mobilized the Δ*recA* mutation into the Δ*dinG* (BG1671) background by SPP1-mediated transduction (Table 1). Since we can move the Δ*recA* mutation into the *pcrA*_T_ cassettes and the Δ*rnhB* or Δ*dinG* background, we tentatively considered that Δ*rnhC* is synthetically lethal in the Δ*recA* context. It is conceivable that RecA prevents R-loop formation and RnhC contributes to their removal, and, in their absence, toxic intermediates accumulate. In *E. coli*, however, Δ*rnhA* Δ*recA* cells are viable [73].

The RecA mediator proteins (RecO and RecR) promote RecA assembly on the ssDNA [22,23]. The role of the RecO mediator, which acts before RecA nucleation on the SsbA-coated ssDNA, is well-characterized in *B. subtilis* [74]. We have tried to move a Δ*recO* mutation into the Δ*rnhC*, and as control in the Δ*rnhB* background by SPP1-mediated transduction and natural chromosomal transformation. We could construct the Δ*recO* Δ*rnhB* strain (Table 1, BG1757). Despite our effort, we could not mobilize the Δ*recO* mutation into the Δ*rnhC* background or the Δ*rnhC* mutation into the Δ*recO* context. It is likely that: (i) *recO* or *recA* inactivation leads to an increased accumulation of intermediates, that should be lethal in the Δ*rnhC*, but not in the Δ*rnhB* context; and (ii) RecA and RnhC aid to overcome RTCs. This is consistent with the observation that BRCA2 (counterpart of bacterial RecO) and Rad51 (counterpart of bacterial RecA) prevent R-loop accumulation [75].

Since RnhC degrades the RNA from the R-loop (see Section 3.1) and PcrA inviability requires RecA [17], but not RnhC (Figure 1), we can envision that PcrA might unwind RNA-DNA hybrids substrates. To test this hypothesis, PcrA was purified and biochemically characterized.

### 3.7. PcrA Preferentially Binds ssDNA and RNA

**Figure 3:**
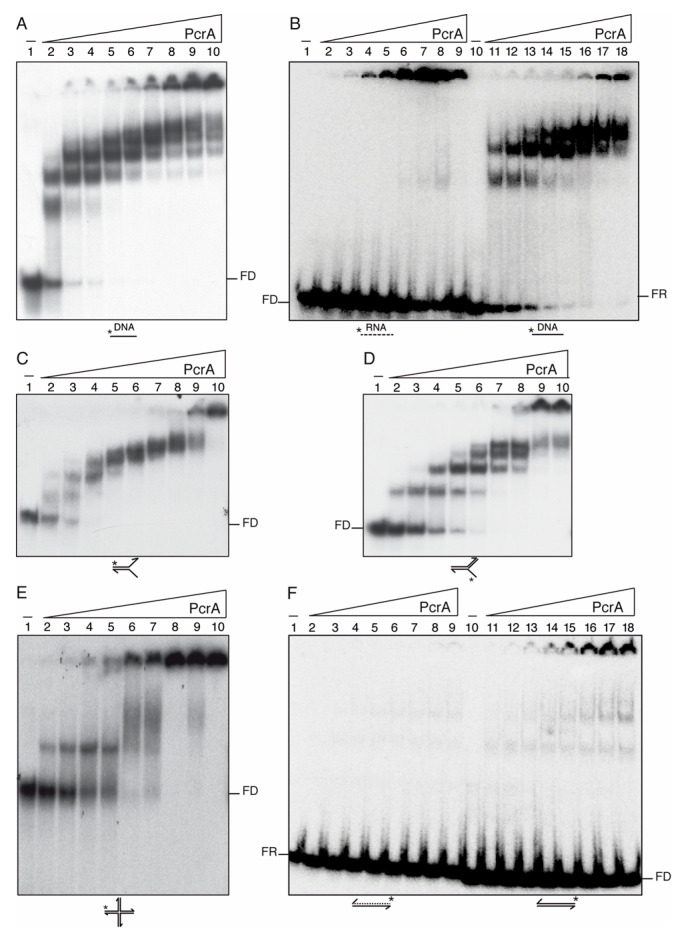
PcrA preferentially binds ssDNA and to a less extent RNA. Increasing concentrations of PcrA (3 to 400 nM (**A**,**B**,**E**,**F**) or 0.3 to 100 (**C**,**D**)) were incubated with a 100-nt long [γ^32^P]-ssDNA (**A**), 38-nt long [γ^32^P]-RNA or ssDNA (**B**), 60-nt long [γ^32^P]-flayed DNA (30-nt tails and 30-bp duplex) (**C**), [γ^32^P]-3′-fork DNA (30-nt 3′-tail, and 30-bp duplex) (**D**), [γ^32^P]-HJ3 DNA (**E**), and 38-bp long [γ^32^P]-duplex DNA or RNA (**F**) in buffer D containing 2.5 mM ATPγS (15 min, 37 °C). Protein-DNA complexes were analyzed by 5% PAGE in 0.25× TBE buffer and autoradiography. K_Dapp_ values were obtained from EMSA assays after electrophoresis. A straight line represents DNA and a dotted line RNA. Abbreviation, FD, free-DNA; FR, free-RNA; * denotes the [γ^32^P]-labeled strand.

To further understand the role of PcrA, the *wt* PcrA protein and its Walker A box mutant (K37A) variant were purified using a similar protocol (Section 2). First, to test whether PcrA binds to different DNA or RNA substrates (Supplemental Figure S1), EMSAs were performed in buffer D containing ATPγS, and the reaction separated in PAGE (see Section 2) (Figure 3A–F).

PcrA bound the 100-nt long [γ^32^P]-ssDNA with an apparent binding constant [K_app_]) of ~1.5 nM (Figure 3A, lanes 3–10). Similar results were observed with *B. stearothermophilus* PcrA (PcrA*_Bst_*) [15]. In contrast, His-tagged PcrA*_Sau_* does not form stable complexes with native ssDNA [76]. This effect could be attributed either to the use of poly(dT) DNA instead of native ssDNA or of a His-tag.

PcrA efficiently bound to a 100-nt long ssDNA or a flayed substrate forming >10 different complexes, with the largest ones not entering into the gel (Figure 3A,C, lanes 2–10). When the size of the ssDNA was reduced to 38-nt, the number of PcrA-ssDNA complexes was also reduced to four different complexes (Figure 3B, lanes 11–18). These results suggest that the number of PcrA monomers on the ssDNA could be related to the length of the substrate with an average size site of 9–7-nt (see Figure 3B–D). This is consistent with the observation that a PcrA*_Bst_* monomer binds to ~8-nt [15]. When *wt* PcrA was replaced by PcrA K37A, similar results to those previously described for the Walker A mutant variant PcrA*_Bst_* K37A were observed (Figure S3A,B) [15].

Then, it was tested whether PcrA bound RNA substrates. PcrA (3–400 nM) bound to the 38-nt [γ^32^P]-RNA with significant lower affinity (K_app_ of ~35 nM) than to ssDNA (Figure 3B, lanes 2–9 vs. 11–18). In contrast, UvrD*_Eco_* does not form a stable complex with an RNA substrate [77]. The formed PcrA-RNA complexes, which were not entering into the gel, were unstable, and free [γ^32^P]-RNA was observed even in the presence of a large PcrA excess (Figure 3B, lane 9). Alternatively, the RNA substrate was folded in a way that PcrA could not form stable complexes with a duplex substrate. To test this hypothesis, the [γ^32^P]-dsDNA or [γ^32^P]-RNA-DNA hybrid substrate was incubated with increasing PcrA concentrations. PcrA failed to form a stable complex with the [γ^32^P]-RNA-DNA hybrid, and large PcrA concentrations were needed to detect [γ^32^P]-dsDNA-PcrA complexes that were not entering into the gel (Figure 3F, lanes 2–9 and 11–18).

A mobile HJ DNA has four duplex arms with a central ssDNA region at the junction. In our case, the HJ4 has 12-nt in the ssDNA form as judged by its resistance to DNase I attack. When the [γ^32^P]-HJ4 DNA was incubated with increasing PcrA concentrations, a single complex shifted to a diffused band and then to a large complex that was not entering into the gel was observed (Figure 3E).

### 3.8. PcrA Is an ssDNA-Dependent ATPase

**Figure 4:**
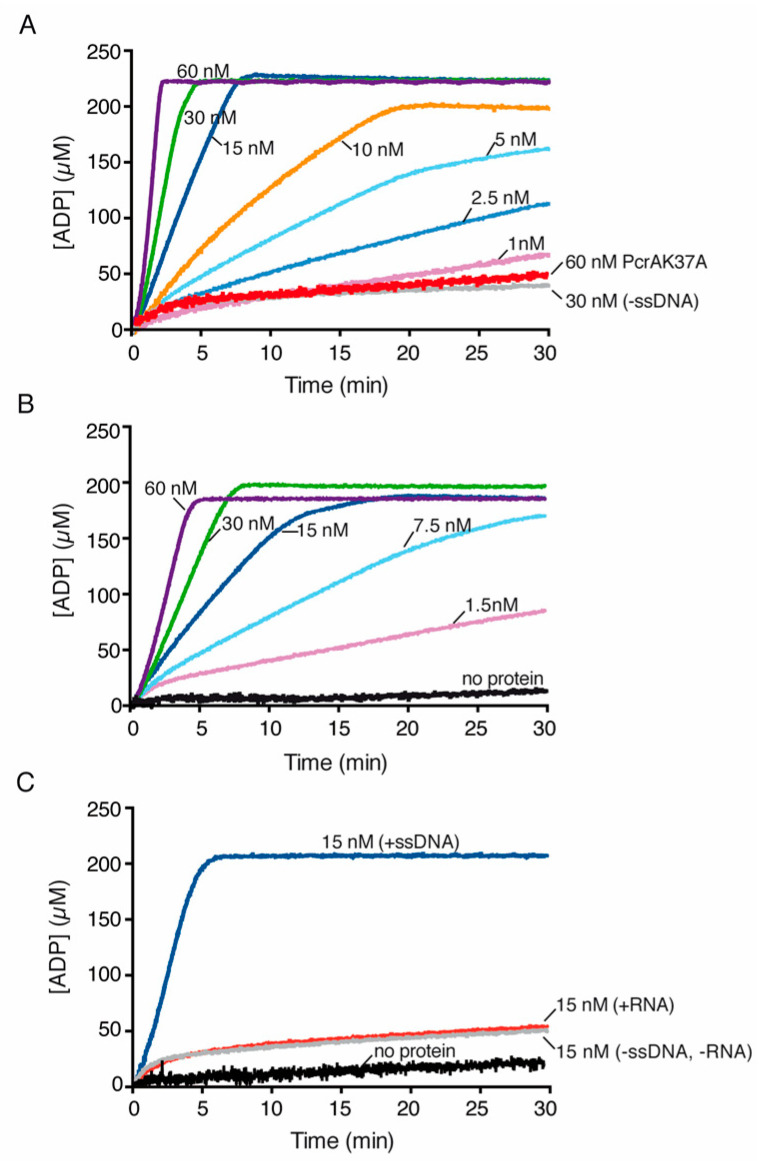
PcrA preferentially hydrolyzes ATP in the presence of ssDNA. PcrA (1–60 nM) was incubated with or without the indicated ssDNA (cssDNA (**A**)), poly(dT) (**B**) or RNA (**C**) at 10 μM (in nts) in buffer E containing 5 mM ATP, and the ATPase activity was measured (30 min, 37 °C). The red line is the ATPase assay of the PcrA K37A mutant variant, the grey line is the control without ssDNA (**A**) and the black line denotes the control reaction corresponding to the ATPase assay in the absence of any protein (**B**,**C**). Representative graphs are shown here, and the determined K_cat_ is shown in Table S1.

To examine whether PcrA hydrolyzes ATP or dATP in the presence of circular 3199-nt pGEM3 Zf(+) ssDNA (cssDNA), the rate of ATP or dATP ([d]ATP) hydrolysis was monitored by analyzing its conversion to ADP or dADP, as described in Section 2. In the absence of PcrA, no ATP/dATP hydrolysis was observed (Figure 4 and Figure S4, black lines). In the absence of DNA, PcrA (15 nM) reached the maximal rate of ATP hydrolysis with a K_cat_ of ~31 min^−^^1^ (Figure 4A, grey line, Table S1).

In the presence of cssDNA, limiting PcrA (1 PcrA monomer/660-nt) strongly stimulated its ATPase activity (by ~60-fold; *p* < 0.01) (Figure 4A, dark blue line, Table S1). Similarly, PcrA*_Bst_* shows a basal level of ATP hydrolysis in the absence of ssDNA and the maximal rate of ATP hydrolysis of PcrA*_Bst_* is significantly stimulated by ssDNA [78,79].

When dATP was provided in place of ATP as the nucleotide cofactor, a biphasic curve of dATP hydrolysis was observed. PcrA-mediated dATP hydrolysis was slower at early times, and then reached the maximal hydrolysis rate. Under these conditions, PcrA hydrolyzed dATP with a ~3 min delay and with a turn-over of dATP significantly slower than that of ATP in the presence of cssDNA (Figure S4A, dark blue line, Table S1).

To test whether the basal PcrA activity in the absence of cssDNA is genuine, PcrA was replaced by the PcrA Walker A box mutant variant (PcrA K37A), which was purified using the same protocol that the one used for the *wt* protein. PcrA K37A (30 or 60 nM) showed a poor ATPase activity, ~60-fold lower than that of the *wt* PcrA (*p* < 0.01) (Figure 4A, red vs. purple lines, Table S1). Similarly, the maximal rate of ATP hydrolysis of PcrA*_Bst_* K37A decreased by ~30-fold when compared with *wt* PcrA*_Bst_* [79].

To confirm the affinity binding predictions, we examined the rate-limiting step(s) within the ATP hydrolysis cycle and performed classic Michaelis–Menten analysis to define the K_m_, K_cat_ and V_max_ (Figure S4B,C). In the presence of variable ATP concentrations as the main substrate (0.06 to 10 mM), limiting PcrA (15 nM) approached a K_m_ for ATP of 1.5 ± 0.3 mM and a V_max_ of ~1875 ± 140 mM·min^−1^ (Figure S4B,C). PcrA*_Bst_* also shows a similar turnover rate and *K*_cat_ (~1500 min^−1^) for ATP, but the K_m_ was ~4-fold smaller in the presence of ssDNA [78,79]. When cssDNA was replaced by unstructured poly(dT) linear ssDNA, the maximal rate of ATP hydrolysis by PcrA was reduced by ~2-fold when compared with cssDNA (Figure 4A,B, Table S1).

When cssDNA was replaced by RNA, the basal PcrA ATPase activity was similar to that in the absence of RNA (Figure 4C, red vs. grey lines, Table S1). The absence of stimulation by RNA cannot be attributed to its degradation during the reaction because we could visualize the RNA substrate after 30 min of incubation (data not shown). Since the maximal rate of ATP hydrolysis by PcrA in the presence of RNA or in the absence of DNA or RNA was similar (Table S1), we assumed that RNA does not stimulate the ATPase activity of PcrA. Similarly, RNA fails to stimulate UvrD*_Eco_*-mediated ATP hydrolysis [77].

### 3.9. PcrA Preferentially Unwinds ssDNA in the 3′→ 5′ Direction

**Figure 5:**
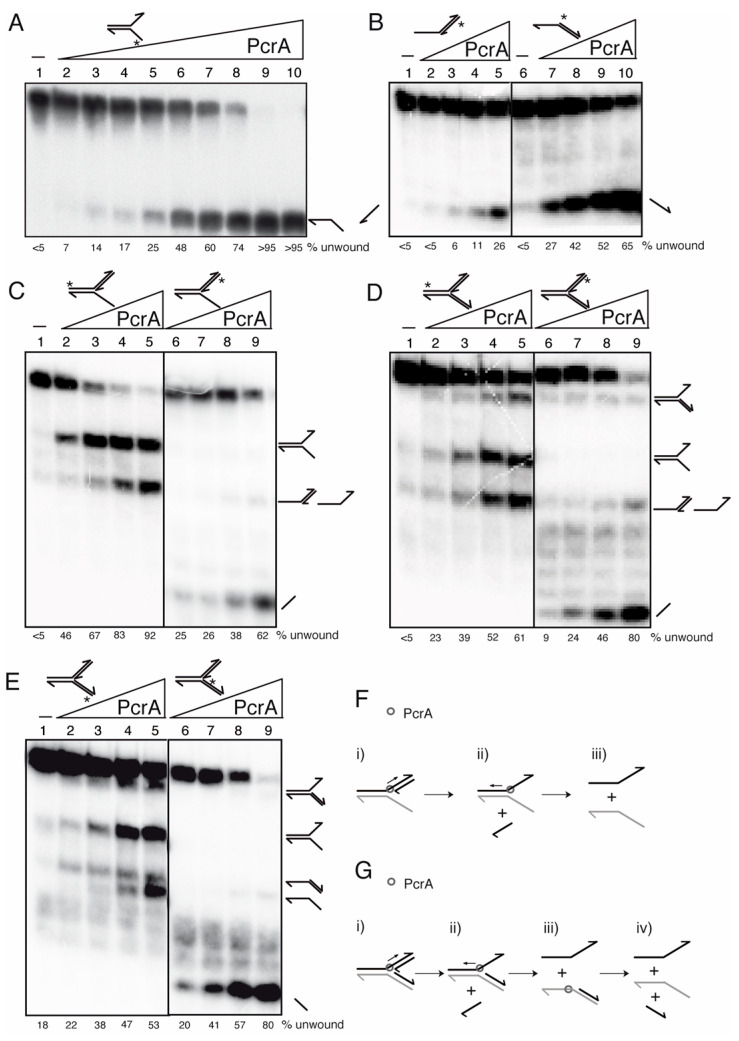
PcrA preferentially unwinds DNA in the 3′→5′ direction. Helicase assays with [γ^32^P]-flayed (**A**), [γ^32^P]-5′- and [γ^32^P]-3′-tailed duplexes (**B**), [γ^32^P]-3′-fork DNA (**C**), and [γ^32^P]-Y-fork (**D**,**E**) were performed with increasing concentrations of *wt* PcrA (0.1–25 nM (**A**) or 1.5 to 12 nM (**B**–**E**)). Reactions were done in buffer D containing 2.5 mM ATP (15 min, 37 °C), and after deproteinization the substrate and products were separated by 6% PAGE in TG buffer, and visualized by phosphor imaging. Cartoons showing the proposed mode of action for PcrA (**F**,**G**). Abbreviations: -, absence of PcrA; *, indicates the [γ^32^P]-labeled strand; half arrow head, denotes the 3′-end.

PcrA*_Bst_* unwinds a DNA substrate in the 3′→5′ direction [78], but a His-tagged PcrA*_Sau_* variant poorly unwinds a flayed DNA substrate with poly(dT) tails, and unwinds a 3′- or 5′-poly(dT) tailed duplex substrate with similar efficiency, suggesting a bipolar unwinding activity of His-tagged PcrA*_Sau_* [76]. To re-evaluate the PcrA helicase activity, the enzyme was incubated in buffer D containing 2.5 mM ATP with different native DNA substrates (Figure 5).

When a [γ^32^P]-flayed DNA substrate (0.25 nM, in DNA molecules) was incubated with increasing PcrA concentrations (0.1 to 25 nM), the unwinding of 50% of the [γ^32^P]-fork DNA substrate was achieved with ~1.5 nM PcrA (Figure 5A, lanes 5–6), suggesting that PcrA efficiently unwinds the flayed DNA (non-replicated fork). PcrA shows a ~9-fold preference for the 3′-tailed duplex substrate (Figure 5B, lanes 5 vs. 7). These data confirmed that PcrA preferentially unwinds a non-replicated fork DNA and a 3′-tailed duplex substrates in the 3′→5′ direction (Figure 5A,B) [78,79].

To re-evaluate whether PcrA preferentially unwinds DNA in the 3′→5′ direction, a non-cognate 3′-fork DNA (a replication fork with a fully synthesized leading-strand and no synthesis in the lagging-strand) in the presence of an excess of the nascent leading strand (to warrant that all the substrate is complexed) was tested (Figure 5C). The 3′-fork DNA, which is an isomer of a D-loop structure, was radiolabeled in the parental leading-strand and was incubated with increasing PcrA (1.5–12 nM) concentrations (Figure 5C, lanes 2–5). PcrA (1.5 nM) at the junction is preferentially bound to the nascent leading-strand to unwind ~50% of the substrate and render a flayed intermediate (Figure 5C, lane 2 and (i) in Figure 5F). In the presence of 12 nM PcrA, the enzyme bound also to the parental leading-strand and unwound the substrate, yielding the flayed and the radiolabeled strand that co-migrate with a [γ^32^P]-5′-tailed duplex (Figure 5C, lanes 4–5). To confirm the above interpretation, the nascent leading-strand was radiolabeled (Figure 5C, lanes 6–9 and (ii) in Figure 5F). PcrA (1.5 nM) bound at the branched point, specifically unwound the [γ^32^P]-3′-nascent leading-strand (Figure 5C, lanes 6–9 and (i) in Figure 5F). Albeit with low efficiency, PcrA (6–12 nM) at the fork junction bound to the template leading-strand and unwound it to generate a [γ^32^P]-5′-tailed duplex (Figure 5C, lanes 8–9).

To test whether PcrA bound at the fork junction preferentially unwinds the nascent leading strand in the 3′→5′ direction, a more complex DNA substrate (a replication fork with both a leading-strand and a lagging-strand at the branch point [Y-fork DNA]) was used (Figure 5D,E). When the parental leading-strand was [γ^32^P]-labeled, it was observed that a higher PcrA concentration (6 nM) was necessary to unwind ~50% of the substrate (Figure 5D, lane 4 and (i) in Figure 5G). PcrA bound at the fork junction accumulated products that co-migrate with a flayed, with a 3′tailed duplex and with the radiolabeled strand (Figure 5D, lanes 4–5 and (i,ii) in Figure 5G). When the radiolabeled strand was the nascent leading-strand one, PcrA bound at the fork junction and displaced the [γ^32^P]-nascent leading-strand (Figure 5D, lanes 6–9 and (iv) in Figure 5G). To re-evaluate these results, the Y-fork DNA was labeled in the parental [γ^32^P]-lagging-strand. PcrA (6 nM) bound at the fork junction displaced both nascent strands, yielding flayed DNA and the free radiolabeled strand (Figure 5E, lane 5). When the labeling was in the nascent [γ^32^P]-lagging-strand, PcrA bound at the fork junction and displaced the nascent [γ^32^P]-lagging-strand (Figure 5E, lane 9, and (iv) in Figure 5G). Alternatively, PcrA entry from the blunt-ended duplex lagging-strands might displace the nascent [γ^32^P]-lagging-strand (see below).

### 3.10. PcrA Unwinds RNA-DNA Hybrids

**Figure 6:**
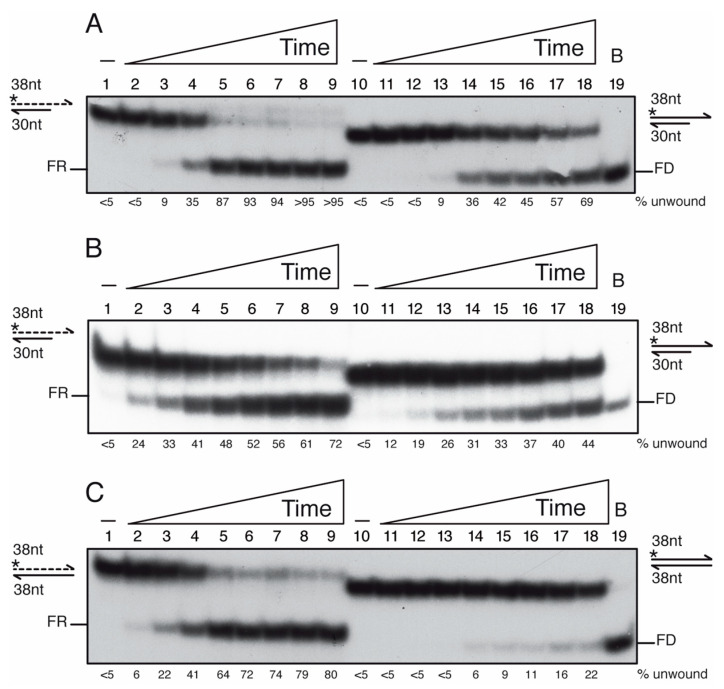
PcrA preferentially unwinds an RNA-DNA hybrid substrate. Helicase assays with [γ^32^P]-3′-tailed duplex DNA or RNA-DNA hybrid (**A**,**B**), or duplex DNA or RNA-DNA hybrid (**C**) and a fixed PcrA concentration (100 nM) varying the incubation time (0.5, 1, 2.5, 5, 7.5, 10, 15 and 20 min, at 37 °C (**A**,**C**) or room temperature (**B**). Reactions were done in buffer D containing 2.5 mM ATP, and, after deproteinization, the substrate and products were separated by 15% PAGE in 0.5× TBE, and visualized by phosphor imaging. Abbreviations: -, the absence of PcrA protein; *, indicates the [γ^32^P]-labeled strand; half arrow head, denotes the 3′-end; B, sample boiled prior loading.

In vivo, the negative outcomes of head-on conflicts are due to pervasive R-loop formation [3,4,5]. In the previous section, we have shown that PcrA unwinds a 3′-tailed duplex with ~9-fold higher efficiency than a 5′-tailed duplex fork substrate (Figure 5B). To test whether monomeric PcrA bound to RNA contributes to unwind an RNA-DNA template, a substrate with a minimal PcrA size site was used (see above). A 38-nt long [γ^32^P]-ssDNA or [γ^32^P]-RNA was annealed to a complementary 30-nt long ssDNA to yield a 3′-tailed DNA duplex or 3′-tailed RNA-DNA hybrid substrate with an 8-nt tail (0.25 nM in DNA molecules) (Figure 6).

When the [γ^32^P]-3′-tailed RNA-DNA hybrid or [γ^32^P]-3′-tailed dsDNA substrate was incubated with increasing PcrA concentrations (0.75 to 100 nM) in buffer D containing 2.5 mM ATP, the unwinding of 50% of the [γ^32^P]-3′-tailed RNA-DNA hybrid substrate was achieved with ~10 nM PcrA and of the [γ^32^P]-3′-tailed duplex DNA substrate was observed at ~45 nM PcrA in a 15 min reaction (Figure S5, lanes 5–6 vs. 17–18). Then, the [γ^32^P]-3′-tailed RNA-DNA hybrid or the [γ^32^P]-3′-tailed dsDNA substrate was incubated with an excess of PcrA (100 nM) for a variable time (0.5, 1, 2.5, 5, 7.5, 10, 15 and 20 min) in buffer D containing 2.5 mM ATP at 37 °C. PcrA unwound ~50% of the [γ^32^P]-3′-tailed RNA-DNA hybrid substrate in ~3 min, but ~12 min were necessary to unwind ~50% of the 3′-tail [γ^32^P]-DNA substrate (Figure 6A, lanes 4–5 vs. 16–17). In 5 min, ~90% of the 3′-tailed [γ^32^P]-RNA hybrid substrate was unwound, but only ~70% of the 3′-tail [γ^32^P]-DNA substrate was unwound in 20 min (Figure 6A, lanes 5–6 vs. 18). Similarly, the UvrD*_Eco_* enzyme unwinds the RNA from the hybrid RNA-DNA substrate with ~6-fold higher efficiency than a tailed-duplex DNA, but here UvrD*_Eco_* binds to circular ssDNA [77].

When this manuscript was submitted, a preprint documenting that PcrA*_Bst_* binds to a 3′-ssDNA tail of an RNA-DNA hybrid and unwinds it at room temperature, but not to a 3′-RNA tail of an RNA-DNA hybrid, was deposited in *bioRxiv* [80]. It is known that the RNA-DNA hybrids adopt a heteromerous conformation, an intermediate between B form dsDNA and A form duplex RNA [3,5,6]. To test the RNA-DNA hybrid “breathing” hypothesis at the end of the duplex, we performed the experiments at room temperature. The [γ^32^P]-3′-tailed RNA-DNA hybrid or the [γ^32^P]-3′-tailed dsDNA substrate was incubated with PcrA for a variable time in buffer containing 2.5 mM ATP (Figure 6B). PcrA unwound ~50% of the [γ^32^P]-3′-tailed RNA-DNA hybrid substrate in ~7 min, but >20 min would be necessary to unwind ~50% of the 3′-tail [γ^32^P]-DNA substrate. This suggests that performing the reaction at room temperature does not alter the outcome, but, as expected, the speed of unwinding was reduced (Figure 6A,B, lanes 4–5 and 16–17 vs. 5–6 and 18).

PcrA binds ssDNA with significant higher affinity than RNA (Figure 3B), thus we asked whether PcrA unwinds an RNA-DNA hybrid substrate without the need of a single-stranded tail. To test the hypothesis, a 38-nt long [γ^32^P]-ssDNA or [γ^32^P]-RNA was annealed to a complementary 38-nt long ssDNA to yield a blunted DNA duplex or hybrid RNA-DNA substrate (0.25 nM in DNA molecules). Unlike UvrD*_Eco_* [81], an excess of PcrA (100 nM) poorly unwound a blunted [γ^32^P]-dsDNA substrate after 20 min incubation (Figure 6C, lane 18). In contrast, PcrA unwound ~50% of the [γ^32^P]-3′-RNA-DNA hybrid substrate in ~3 min at 37 °C (Figure 6C, lane 5). Therefore, it is unclear whether PcrA unwinds the hybrid substrate by binding to the 3′-RNA tail or to 3′-DNA by entering at a blunt end. When ATP was replaced by the non-hydrolysable ATPγS analog, PcrA neither unzips the RNA-DNA hybrid nor the DNA duplex substrate (data not shown). It will be of significant interest to determine the molecular basis of PcrA unwinding of the blunted DNA-RNA hybrid substrate.

## 4. Discussion

The phenotypes associated with PcrA depletion are complex and reflect its involvement in several DNA transactions. The present work leads to eight main proposals. First, our results show that, in the presence of mis-incorporated rNMPs (or in the absence of RnhB), the endogenous threats generated by PcrA depletion were significantly reduced. It is unknown whether the lack of DNA nicks at rNMPs (in the Δ*rnhB* context) or the presence of a rNMPs mis-insertion contributes to cell viability upon PcrA depletion. Since the presence of random nicks or abasic sites, as those induced by H_2_O_2_ or MMS exposure, neither reduces survival nor compromises the degree of suppression upon PcrA depletion in the Δ*rnhB* context, we favor the hypothesis that the presence of rNMPs mis-insertions on the DNA template delays DNAP, halts RNAP movement [60,61], suppresses PcrA lethality, and indirectly provides more time for the removal of RTCs and for their repair by specialized or general homologous recombination pathways.

Second, in the absence of the primary RER mechanism (i.e., in the Δ*rnhB* context), the mis-incorporated rNMPs, which induce changes in the structure and conformation of DNA, can be efficiently recognized by the RNAP, exposed to PcrA- [or UvrD*_Eco_*]-mediated RNAP backtracking, and the offending distortion removed by the UvrBC complex [49,62,64,82]. Upon PcrA depletion, the mis-incorporated rNMPs are removed via global-genome NER or Mfd-dependent TCR sub-pathway or repair-by-recombination mechanisms in the Δ*rnhB* context [49,62].

Third, PcrA indirectly helps to remove the non-bulky lesions via repair-by-recombination mechanisms. This assumption is supported by the fact that PcrA depletion lethality is suppressed by *recA* inactivation, but the survival of the Δ*recA pcrA*_T_ strain to IPTG and limiting MMS [17] or 4NQO concentrations (data not shown) was extremely reduced, to levels comparable to that of the most sensitive Δ*recA* strain.

**Figure 7:**
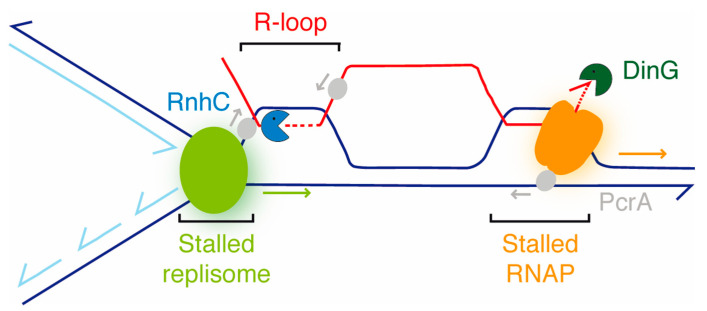
Accessory RNAP modulators (nucleases [RnhC, DinG] and a DNA helicase [PcrA]) aid in the resolution of RTCs. In a co-directional model, the RNAP (orange) stalls at a bulky lesion and impedes DNAP (light green) progression, and an R-loop is formed at the RTC. PcrA (gray) translocating in the non-template strand in the 3′→5′ direction interacts with the RNAP and backtracks it, with DinG (dark green) degrading the exposed 3′-end of the mRNA to facilitate transcription re-initiation if the lesions were removed. RnhC (blue), upon interacting with the RNAP, cleaves the RNA at the R-loop, and then PcrA unwinds it from the DNA-RNA hybrid. The arrows denote the direction of the indicated enzyme.

Fourth, PcrA, in concert with RnhC or DinG, contribute to overcome RTCs, but RnhC acts via a different mechanism to that of DinG (Figure 1, Figure 2 and Figure 7). Since *dinG* inactivation does not lead to the accumulation of unresolved branched toxic intermediates upon PcrA depletion (see Figure 2), we have to assume that PcrA may not process reversed forks. Indeed, PcrA is unable to regress a reversed fork (a HJ-like structure), to render two flayed DNA products (B.C. personal communication). Alternatively, the role of PcrA on RTCs is via the removal of RecA bound to the ssDNA region at potential R-loops. Since there are controversies in the literature, namely a PcrA variant blocked in translocase/helicase activity (PcrA K37A Q254A) that is unable to alleviate RCTs [12] while an equivalent variant (PcrA*_Sau_* K33A Q250R) can remove RecA nucleoprotein filaments from ssDNA and prevent RecA-mediated DNA strand exchange [29], we will address the anti-recombinase role of PcrA elsewhere.

Fifth, PcrA contributes to disassembly RTCs, because PcrA depletion lethality is significantly enhanced by *rnhC* or *dinG* inactivation (Figure 1), suggesting that an endogenous source of genome instability (e.g., RTCs) significantly increases cell death. However, the sensitivity of the assay does not allow us to evaluate whether PcrA works in concert with RnhC or DinG in response to exogenous genotoxic agents. We consider unlikely that the *pcrA* gene is epistatic to *rnhC* and *dinG* in response to DNA damage, and PcrA depletion lethality accumulates different types of recombination intermediates in the *rnhC* to those accumulated in the Δ*dinG* background (Figure 1 and Figure 2).

Sixth, PcrA (or UvrD*_Eco_*) interacts with a stalled RNAP [31,32,81,82,83], backtracks it and exposes the 3′-end of the mRNA. Then, PcrA counteracts the accumulation of branched intermediates (Figure 2) and disassembles RNA-DNA hybrids (Figure 6), which accumulate in the Δ*rnhC* context as proposed in Figure 7. RnhC, which interacts with a stalled RNAP [31], cleaves the RNA at the RNA-DNA hybrid substrate (Figure 7) [84]. Then, PcrA bound to the ssDNA may dismantle the RNA-DNA hybrids to counteract the accumulation of branched intermediates in the absence of both PcrA and RnhC (Figure 2 and Figure 7). In contrast, in *E. coli* cells, SSB interacts with and contributes to RnhA-mediated removal of transcription-dependent R-loop obstacles by localizing the enzyme to the stalled replication fork [71].

Seventh, *recA* or *recO* inactivation is synthetically lethal in the Δ*rnhC* context, suggesting that RecO and RecA may prevent R-loop formation, as proposed for the human Rad51 and its mediator BRCA2 [75], and RnhC (counterpart of RnhA*_Eco_*) degrades R-loops [2,4,5,33]. This assumption is based on the fact that: (a) PcrA lethality is significantly enhanced by *rnhC* inactivation (Figure 1), and inactivation of functions that promote the disassembly of RecA nucleoprotein filaments, as RecX or RecU, significantly reduced cell viability upon PcrA depletion [17]; (b) PcrA depletion lethality is suppressed by *recA* inactivation [17]; (c) PcrA depletion blocks chromosomal segregation in the Δ*rnhC* or Δ*recA* context (Figure 2); and (d) PcrA preferentially catalyzes the unwinding of 3′-tailed RNA-DNA hybrid substrates when compared to 3′-tailed DNA (Figure 6). In contrast, RecA*_Eco_* or Rad51*_Sce_* contributes to R-loop formation through an inverse strand exchange reaction [24,25,73] and RnhA degrades them [33]. Indeed, *E. coli* Δ*recA* Δ*rnhA* or yeast Δ*rad51* Δ*rnh1* Δ*rnh201* mutant cells are viable [73,82]. Finally, PcrA backtracks the stalled RNAP to expose the last incorporated ribonucleotide, and the DinG exonuclease bound to the 3′-end of the mRNA degrades it (Figure 6), as earlier proposed [53]. After damage repair, RNAP elongation can reactivate. Then, replication restart is facilitated upon reactivation of RNAP elongation.

In summary, our results agree with a model in which PcrA contributes to the removal of RTCs. We show that PcrA efficiently unwinds RNA-DNA hybrids, and propose that PcrA and RnhC promote the removal of branched intermediates and RTCs by a different mechanism to that mediated by PcrA and DinG. The potential contribution of the accessory PcrA DNA helicase in promoting replisome movement through nucleoprotein barriers will be addressed elsewhere.

## Figures and Tables

**Table 1:** Strains and plasmid.

**Strains ^a^**	**Relevant Genotype**	**Source/Reference**
BG214	*wt*	Laboratory strain
BG1525	+*pcrA-ssrA sspB* (*pcrA*_T_)	[37]
BG1873	+∆*recA*	[17]
BG1877	+*pcrA*_T_ ∆*recA*	[17]
BG1711	+*pcrA*_T_ ∆*recU*	[17]
BG1749	+∆*rnhB*	This work
BG1751	+∆*rnhC*	This work
BG1605	+∆*dinG*	This work
BG1867	+*pcrA*_T_ ∆*dinG*	This work
BG1863	+*pcrA*_T_ ∆*rnhB*	This work
BG1865	+*pcrA*_T_ ∆*rnhC*	This work
BG1753	+∆*rnhB* ∆*recA*	This work
BG1757	+∆*rnhB* ∆*recO*	This work
BG1671	+∆*dinG* ∆*recA*	This work
**Plasmid ^b^**	**Relevant Genotype**	**Source/Reference**
pCB1229	+*pcrA*, Amp^R^, *ori_Eco_*	[17]
pCB1230	+*pcrA* K37A, Amp^R^, *ori_Eco_*	[17]

^a^ All *B. subtilis* strains are derivatives of the BG214 (*trpCE metA*5 *amyE1 ytsJ*1 *rsbV*37 *xre*1 *xkd*A1 *att*^SPß^*att*^ICE*Bs*1^) strain. ^b^ The plasmid-borne *pcrA* gene and its variant were used to overexpress *B. subtilis* PcrA and PcrA K37A in the heterologous *E. coli* M15 (pREP4) (QIAexpress) host.

## Data Availability

Datasets were generated during the study. We endorsed MDPI Research Data Policies.

## Supplementary Materials

The following are available online at https://www.mdpi.com/article/10.3390/cells10040935/s1, Table S1: ssDNA-dependence of PcrA-mediated ATP hydrolysis, Figure S1: Scheme and composition of the DNA substrates used in this study. Figure S2: Survival of *wt* and single and double mutants to cytotoxic agents, Figure S3: PcrA K37A binds ssDNA, but poorly does to RNA, Figure S4: PcrA hydrolyzes dATP with lower efficiency, Figure S5: PcrA preferentially unwinds 3′-tailed RNA-DNA hybrids.

## Note Added in Proof

When this manuscript was submitted, a preprint evaluating a role for PcrA in the suppression of R-loops via an interaction with the RNAP was deposited by Urrutia-Irazabal and co-workers in *bioRxiv* (doi:10.1101/2021.03.15.435481).
