# Supplementary material for: Bacillus subtilis PcrA Helicase Removes Trafficking Barriers
Source: Cells. 2021 Apr 17;10(4):935. doi: 10.3390/cells10040935 (PMC8074105; doi:10.3390/cells10040935)
Supplement: Supplementary file 1 [file cells-10-00935-s001.zip › cells-1164145-supplementary.pdf]

Bacillus subtilis **PcrA helicase removes trafficking barriers**

María Moreno-del Álamo†, Begoña Carrasco†, Rubén Torres, and Juan Carlos Alonso*

Department of Microbial Biotechnology, Centro Nacional de Biotecnología, CNB-CSIC, 28049 Madrid, Spain

†These authors contributed equally to this work

Table S1. ssDNA-dependence of PcrA ATP hydrolysis

Proteins	K_{cat} (min^{-1})					
	ATP (5 mM)				dATP (5 mM)	
	cssDNA[b]	poly(dT)[b]	RNA[b]	none	cssDNA[b]	none
PcrA (15 nM)	1790 ± 379	915 ± 106	33 ± 3	31 ± 5.2	639 ± 131	30 ± 5.0
PcrAK37A (15 nM)	26.5 ± 5.0	-	-	35 ± 4.8	-	-

[a]The rate of PcrA-mediated ATP hydrolysis was measured as described (see Materials and methods) and were derived from more than three independent experiments like those shown in Figure 4 and S4. [b]The substrates (circular ssDNA [cssDNA], poly(dT) DNA [poly(dT)], or RNA) are 3,199-nt, 20-nt and 38-nt long. The results are shown as mean ± SEM. The - symbol indicates not done.

DNA	Substrate description	Structure + oligonucleotide composition
ssDNA	100-nt / 38nt ssDNA	Fork1/SM44D 100/38
ssRNA	38nt ssRNA	SM44R 38
3'-tailed	3'-tailed DNA	173 30 30 172
5'-tailed	5'-tailed DNA	170 30 30 171
flayed	forked DNA	170 30 30 30 173
3'-fork	3'-forked DNA	170 30 30 171 30 173
Y-fork	Replicated-fork DNA	170 30 30 171 173 30 172
HJ4	mobile Holliday junction	170 173 345 346
dsDNA	38-bp dsDNA	SM44D 38 38 SM45D
3'-short-tailed	3'-tailed DNA	SM44D 30 8 SM46D
5'-short-tailed	5'-tailed DNA	SM44D 30 8 SM47D
RNA-DNA hybrid	38-bp dsRNA-DNA	SM44R 38 38 SM45D
3'-RNA-DNA hybrid	3'-tailed RNA-DNA	SM44R 30 8 SM46D
5'-RNA-DNA hybrid	5'-tailed RNA-DNA	SM44R 30 8 SM47D

Fig. S1. Scheme and composition of the DNA substrates used in this study. In dotted lines is denoted the RNA, and with straight lines the DNA. The non-complementary strands are denoted in grey and the complementary ones in black

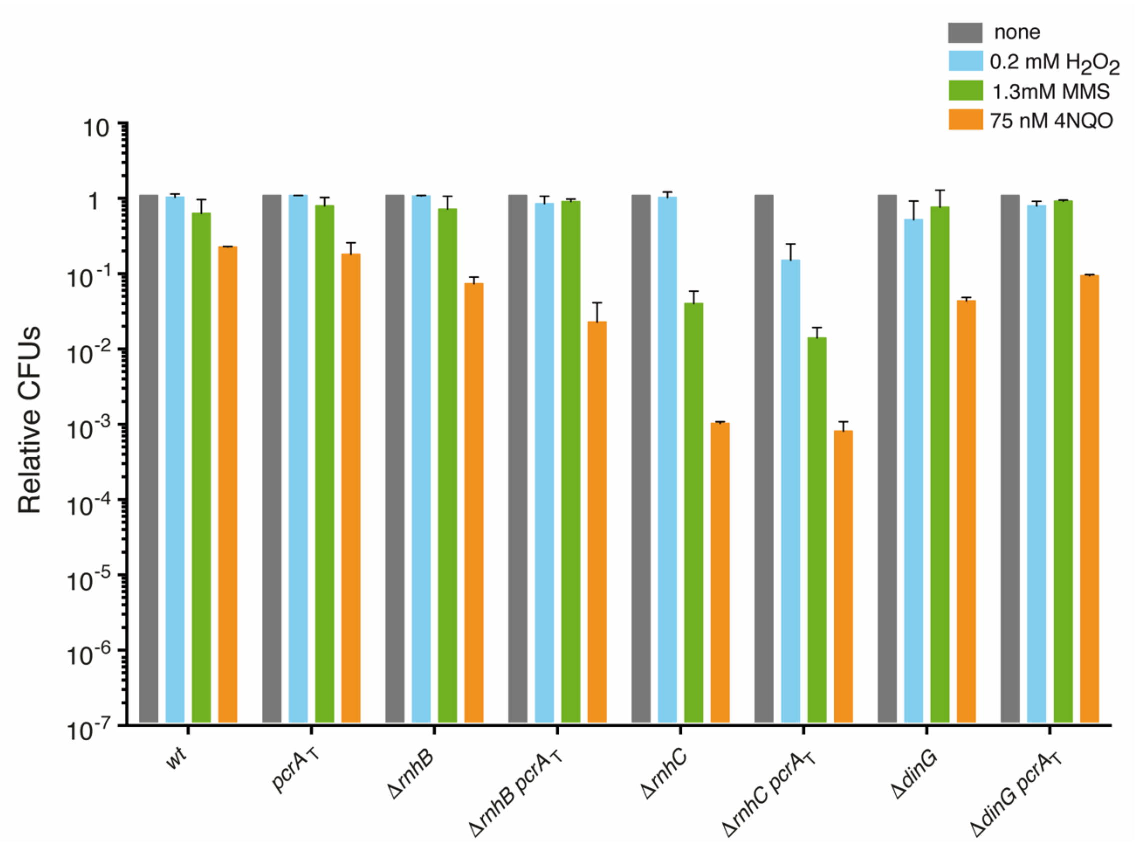

Fig. S2. Survival of *wt* and single and double mutants to cytotoxic agents. Log phase cultures of *wt*, single (*pcrA*T, *rnhB*, *rnhC* or *dinG*) and double mutant (*pcrA*T *rnhB*, *pcrA*T *rnhC* or *pcrA*T *dinG*) strains were diluted, plated on LB agar containing H_2O_2, MMS or 4NQO or lacking the clastogens (dark grey bars), and incubated overnight at 37 ºC. Experiments were performed at least four times. Data are shown as mean fractional survival ± SEM.

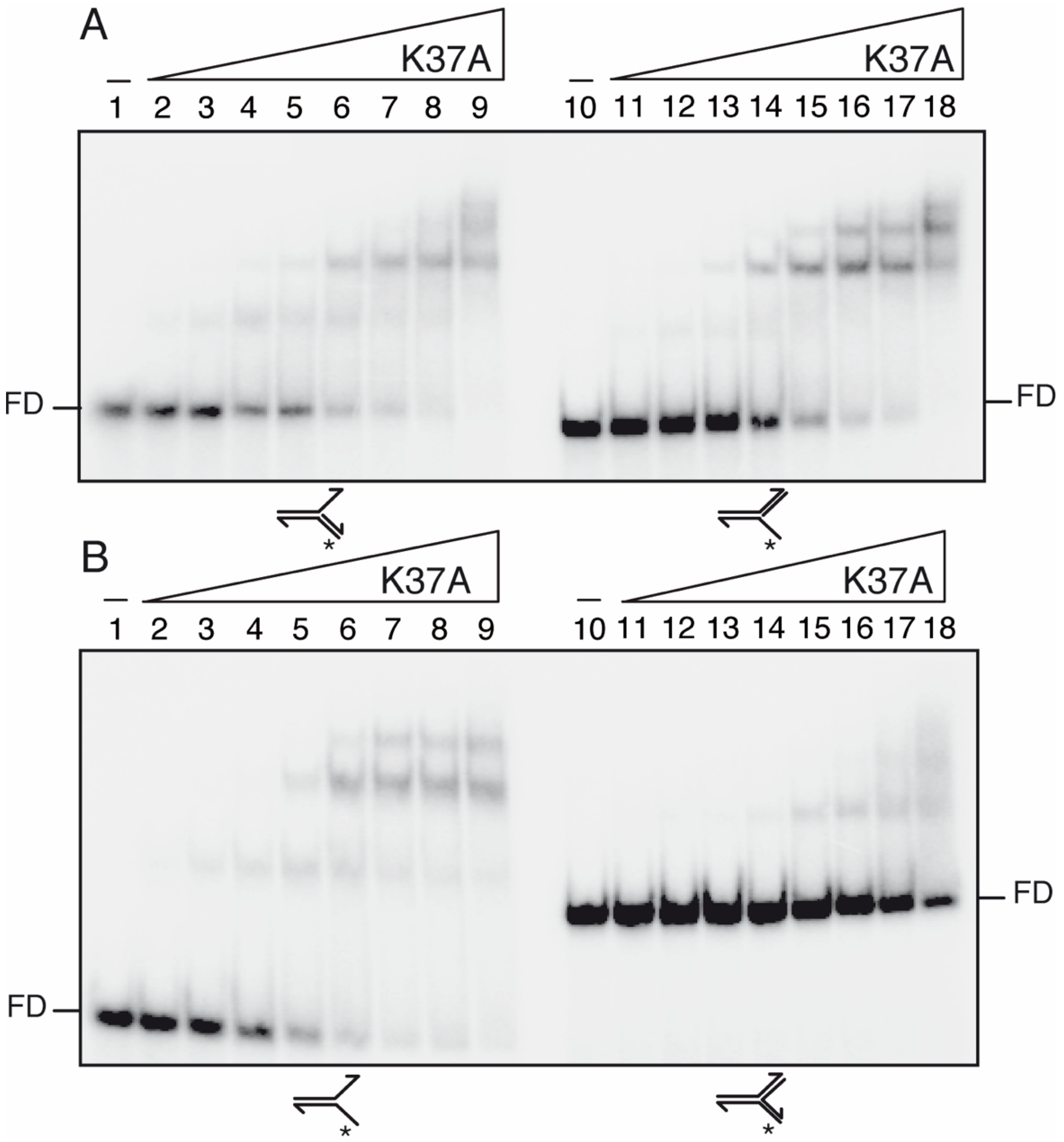

Fig. S3. PcrA K37A binds ssDNA, but poorly does to RNA. Increasing concentrations of PcrA K37A (0.1 to 25 nM) were incubated with [γ^{32}P]-5′or [γ^{32}P]-3′-fork DNA (A) or [γ^{32}P]-flayed DNA and [γ^{32}P]-Y-fork DNA (B) in buffer D containing 2.5 mM ATPγS substrate and products were separated by 5% PAGE in 0.25X TBE buffer. K_{Dapp} values were obtained from EMSA assays after electrophoresis.

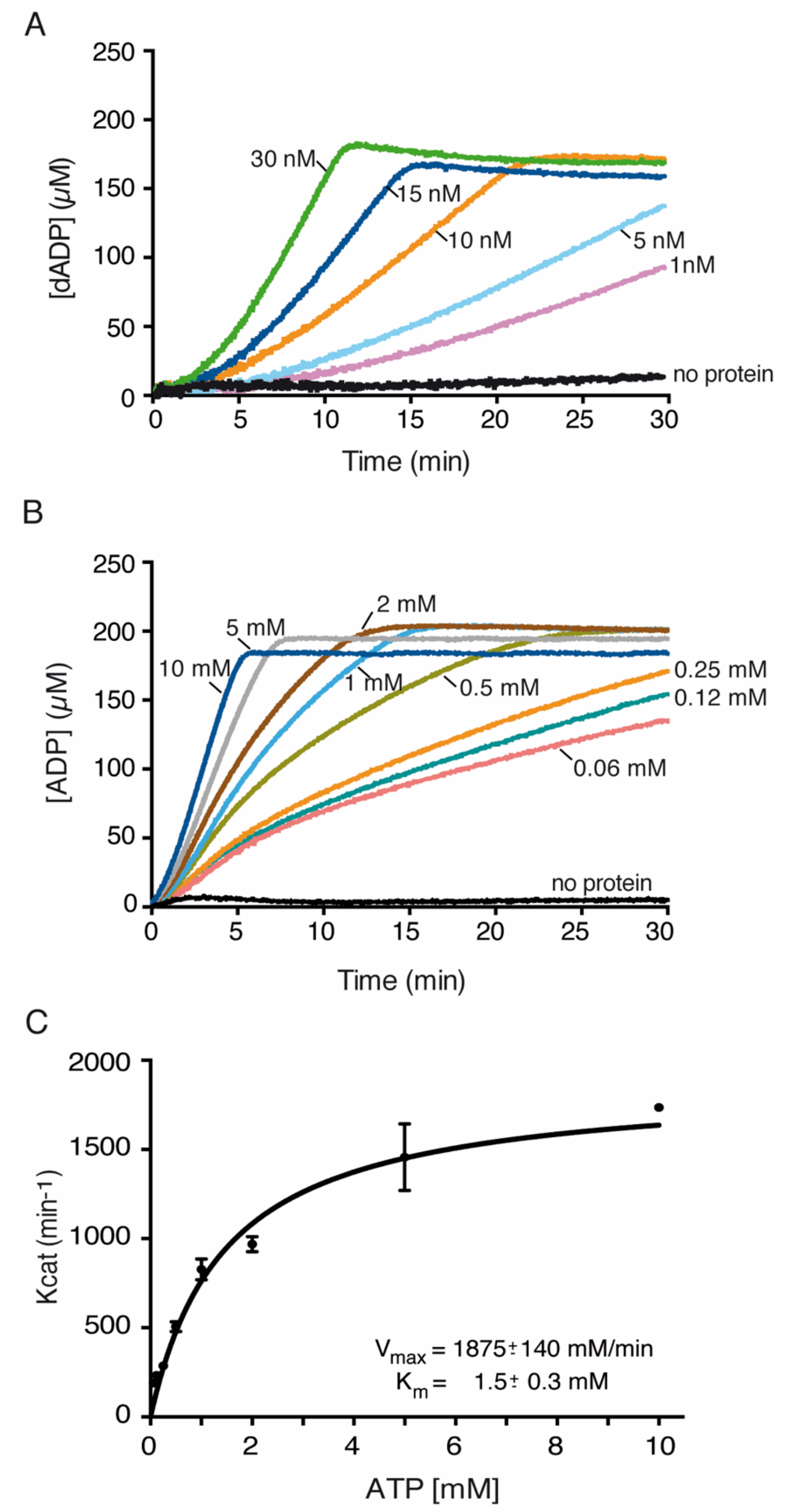

Fig. S4. PcrA hydrolyzes dATP with lower efficiency than ATP. (A) PcrA (1 - 30 nM) was incubated with or without cssDNA at 10 μM (in nts) in buffer E containing 5 mM dATP and the dATPase activity was measured (30 min, 37 ºC). The black line denotes the control reaction corresponding to an dATPase assay in the absence of PcrA. (B) PcrA (15 nM) was incubated with cssDNA at 10 μM (in nts) in buffer A containing increasing ATP concentrations and the ATPase activity was measured (30 min, 37 ºC). (A-B) Representative graphs are shown here. (C) Steady-state ATPase activity of PcrA. The measurements were carried out at 37 ºC in buffer A containing increasing ATP concentrations. The line is the one that best fits to the Michaelis-Menten equation to give a Km of 1.5 μM and a V_{max} of 1875 mM min^{-1}.

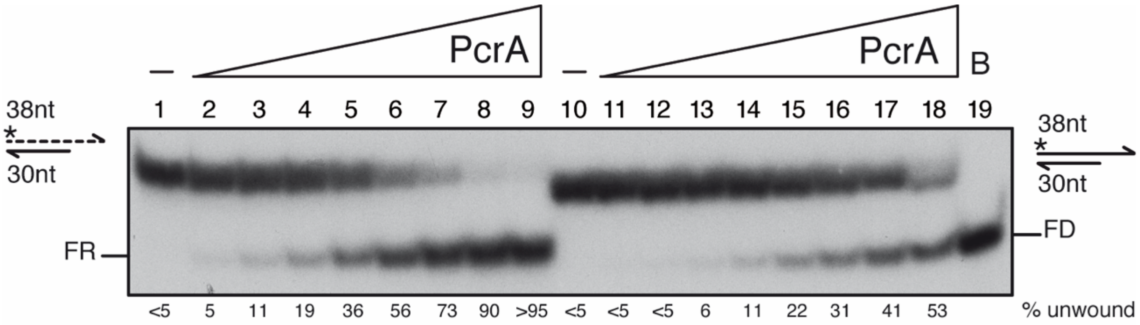

Fig. S5. PcrA preferentially unwinds 3´-tailed RNA-DNA hybrids. Helicase assays with short [γ^{32}P]-3´-tailed RNA-DNA hybrids (lines 2-9) or [γ^{32}P]-3´-tailed duplex (lines 11-18) in the presence of increasing PcrA concentrations (0.7 to 100 nM) (15 min, 37 ºC) in buffer D containing 2.5 mM ATP. After deproteinization, the substrate and products were separated by 6% PAGE and visualized by phosphor imaging. Abbreviations: - and +, the absence or presence of PcrA protein; *, indicates the [γ^{32}P]-labeled strand; half arrow denotes the 3´-end; B, sample boiled prior loading.

Bacillus subtilis PcrA helicase removes trafficking barriers

María Moreno-del Álamo†, Begoña Carrasco†, Rubén Torres, and Juan Carlos Alonso*

Department of Microbial Biotechnology, Centro Nacional de Biotecnología, CNB-CSIC, 28049 Madrid, Spain

†These authors contributed equally to this work

Table S1. ssDNA-dependence of PcrA ATP hydrolysis

Proteins	K_{cat} (min^{-1})					
	ATP (5 mM)				dATP (5 mM)	
	cssDNA[b]	poly(dT)[b]	RNA[b]	none	cssDNA[b]	none
PcrA (15 nM)	1790 ± 379	915 ± 106	33 ± 3	31 ± 5.2	639 ± 131	30 ± 5.0
PcrAK37A (15 nM)	26.5 ± 5.0	-	-	35 ± 4.8	-	-

[a]The rate of PcrA-mediated ATP hydrolysis was measured as described (see Materials and methods) and were derived from more than three independent experiments like those shown in Figure 4 and S4. [b]The substrates (circular ssDNA [cssDNA], poly(dT) DNA [poly(dT)], or RNA) are 3,199-nt, 20-nt and 38-nt long. The results are shown as mean ± SEM. The - symbol indicates not done.

DNA	Substrate description	Structure + oligonucleotide composition
ssDNA	100-nt / 38nt ssDNA	Fork1/SM44D 100/38
ssRNA	38nt ssRNA	SM44R 38
3'-tailed	3'-tailed DNA	173 30 30 172
5'-tailed	5'-tailed DNA	170 30 30 171
flayed	forked DNA	170 30 30 30 173
3'-fork	3'-forked DNA	170 30 30 171 30 173
Y-fork	Replicated-fork DNA	170 173 30 30 171 30 172
HJ4	mobile Holliday junction	170 173 345 346
dsDNA	38-bp dsDNA	SM44D 38 38 SM45D
3'-short-tailed	3'-tailed DNA	SM44D 30 8 SM46D
5'-short-tailed	5'-tailed DNA	SM44D 30 8 SM47D
RNA-DNA hybrid	38-bp dsRNA-DNA	SM44R 38 38 SM45D
3'-RNA-DNA hybrid	3'-tailed RNA-DNA	SM44R 30 8 SM46D
5'-RNA-DNA hybrid	5'-tailed RNA-DNA	SM44R 30 8 SM47D

Fig. S1. Scheme and composition of the DNA substrates used in this study. In dotted lines is denoted the RNA, and with straight lines the DNA. The non-complementary strands are denoted in grey and the complementary ones in black

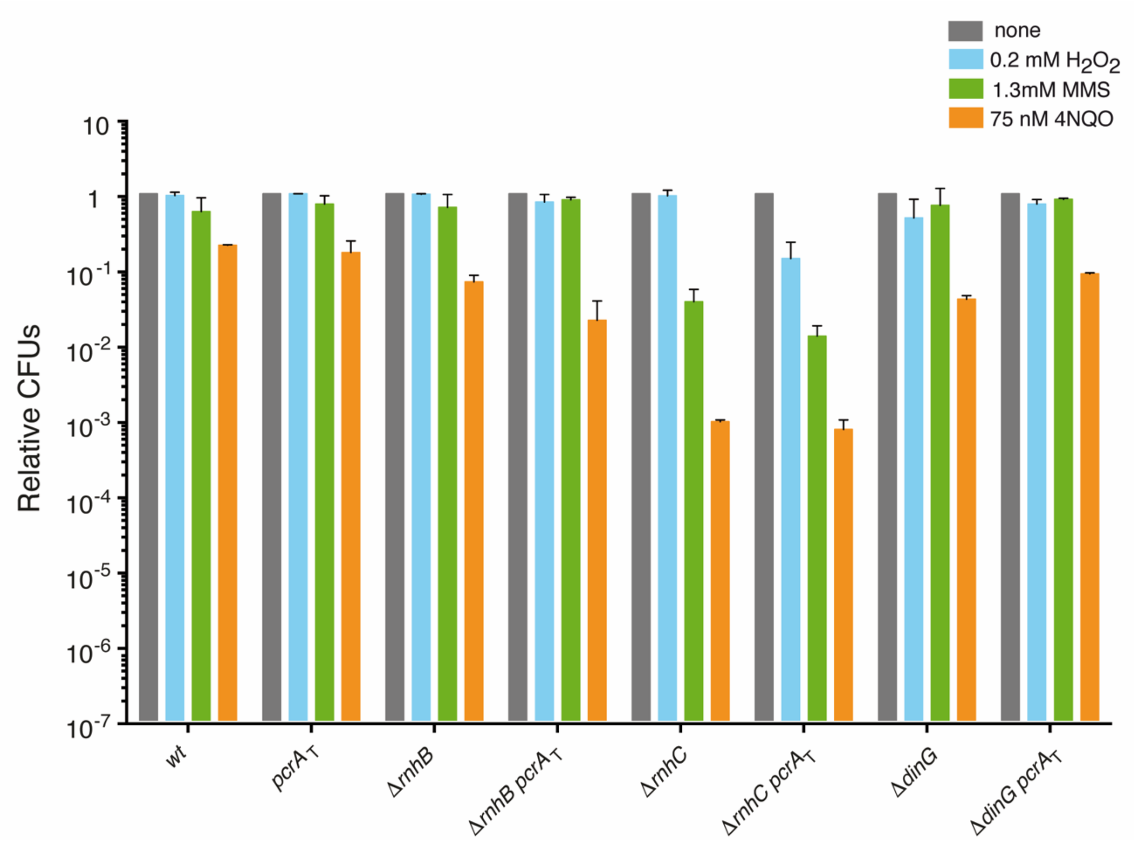

Fig. S2. Survival of *wt* and single and double mutants to cytotoxic agents. Log phase cultures of *wt*, single (*pcrA*T, *rnhB*, *rnhC* or *dinG*) and double mutant (*pcrA*T *rnhB*, *pcrA*T *rnhC* or *pcrA*T *dinG*) strains were diluted, plated on LB agar containing H_2O_2, MMS or 4NQO or lacking the clastogens (dark grey bars), and incubated overnight at 37 ºC. Experiments were performed at least four times. Data are shown as mean fractional survival ± SEM.

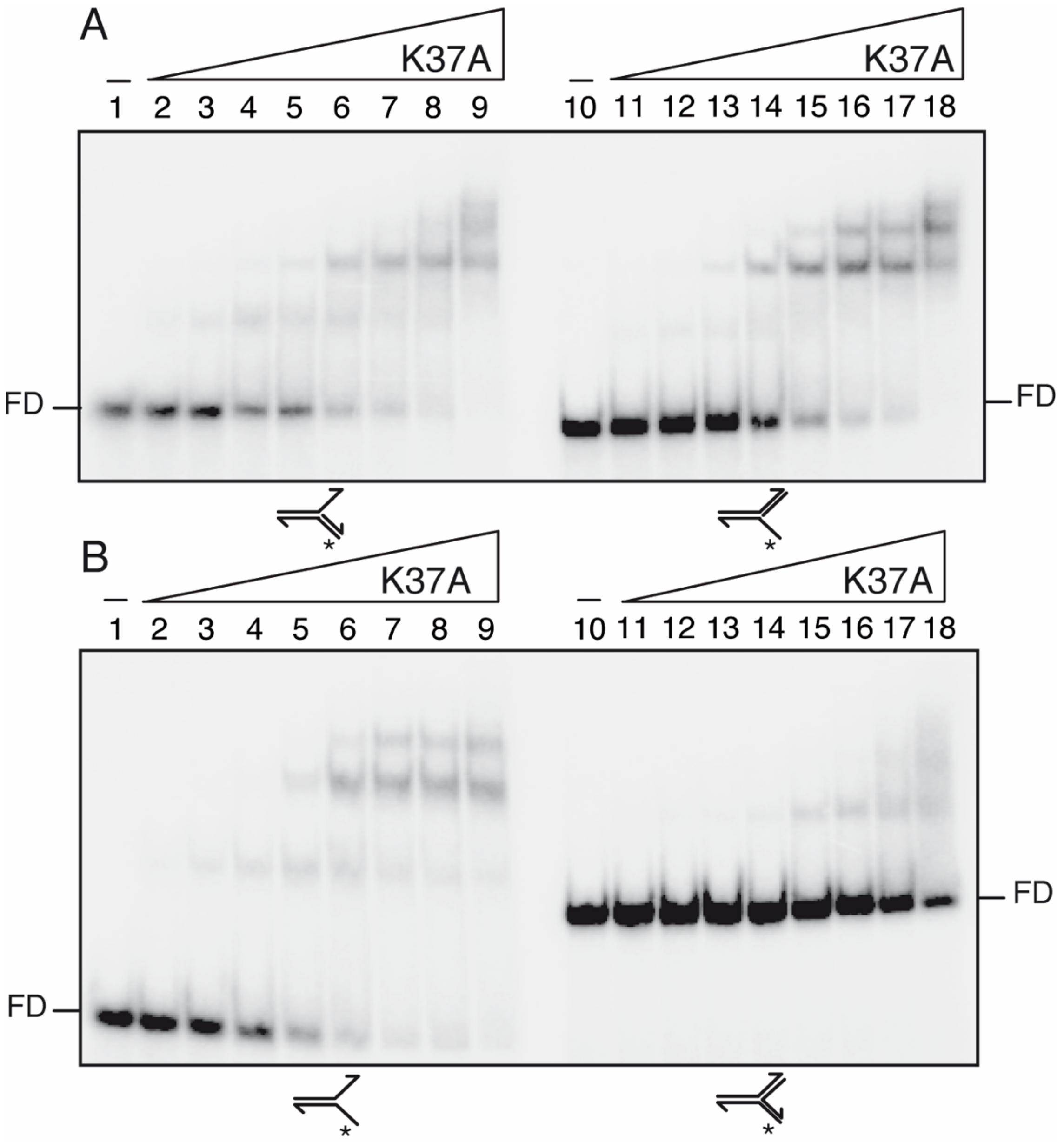

Fig. S3. PcrA K37A binds ssDNA, but poorly does to RNA. Increasing concentrations of PcrA K37A (0.1 to 25 nM) were incubated with [γ^{32}P]-5′or [γ^{32}P]-3′-fork DNA (A) or [γ^{32}P]-flayed DNA and [γ^{32}P]-Y-fork DNA (B) in buffer D containing 2.5 mM ATPγS substrate and products were separated by 5% PAGE in 0.25X TBE buffer. K_{Dapp} values were obtained from EMSA assays after electrophoresis.

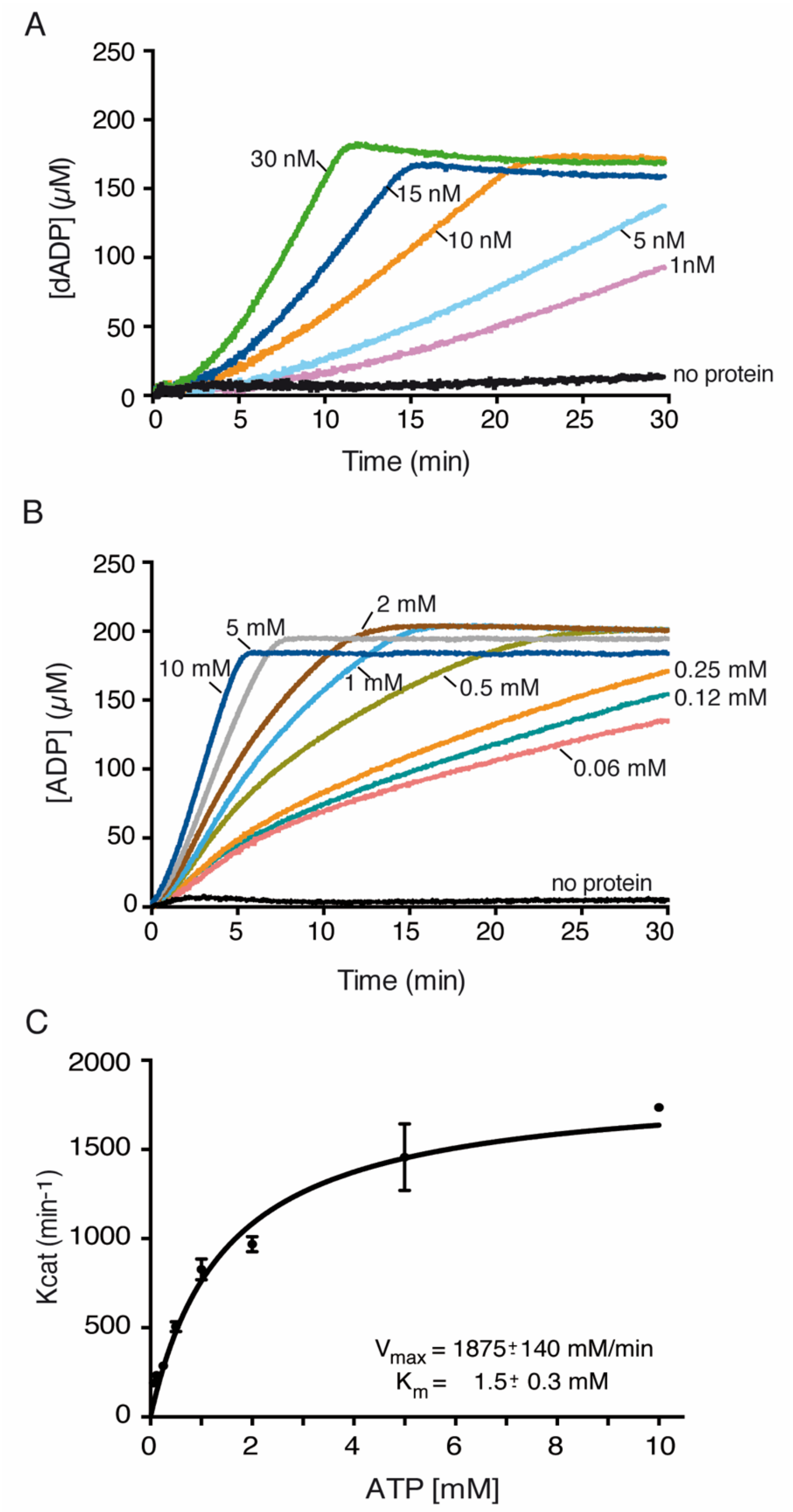

Fig. S4. PcrA hydrolyzes dATP with lower efficiency than ATP. (A) PcrA (1 - 30 nM) was incubated with or without cssDNA at 10 μM (in nts) in buffer E containing 5 mM dATP and the dATPase activity was measured (30 min, 37 ºC). The black line denotes the control reaction corresponding to an dATPase assay in the absence of PcrA. (B) PcrA (15 nM) was incubated with cssDNA at 10 μM (in nts) in buffer A containing increasing ATP concentrations and the ATPase activity was measured (30 min, 37 ºC). (A-B) Representative graphs are shown here. (C) Steady-state ATPase activity of PcrA. The measurements were carried out at 37 ºC in buffer A containing increasing ATP concentrations. The line is the one that best fits to the Michaelis-Menten equation to give a Km of 1.5 μM and a V_{max} of 1875 mM min^{-1}.

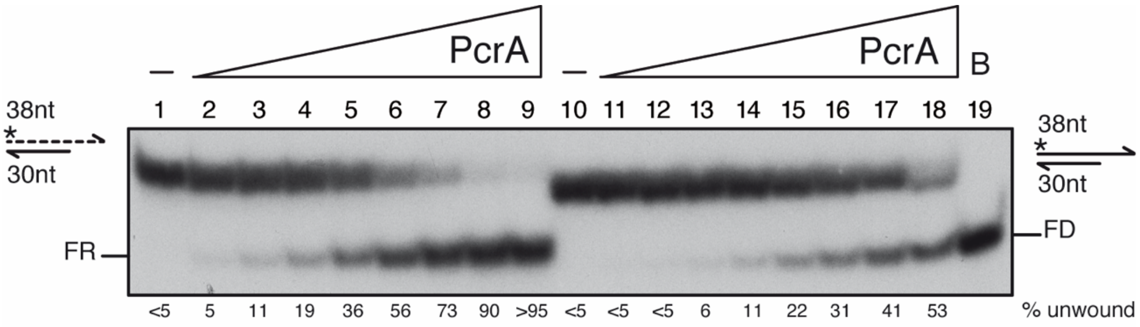

Fig. S5. PcrA preferentially unwinds 3´-tailed RNA-DNA hybrids. Helicase assays with short [γ^{32}P]-3´-tailed RNA-DNA hybrids (lines 2-9) or [γ^{32}P]-3´-tailed duplex (lines 11-18) in the presence of increasing PcrA concentrations (0.7 to 100 nM) (15 min, 37 ºC) in buffer D containing 2.5 mM ATP. After deproteinization, the substrate and products were separated by 6% PAGE and visualized by phosphor imaging. Abbreviations: - and +, the absence or presence of PcrA protein; *, indicates the [γ^{32}P]-labeled strand; half arrow denotes the 3´-end; B, sample boiled prior loading.
